# Human platelet-derived extracellular vesicle fractions modulate bone cell metabolism and biologize volume-stable β-TCP matrix in vitro

**DOI:** 10.1186/s12916-025-04371-w

**Published:** 2025-10-21

**Authors:** Annika Döding, Alexander Güllich, Simon Koch, Kyra de Miroschedji, Ulrike Schulze-Späte

**Affiliations:** 1 Section of Geriodontics, Department of Conservative Dentistry and Periodontology, Center of Dental Medicine, Jena University Hospital, Friedrich Schiller University Jena, Jena, Germany; 2 Lysatpharma GmbH, Eisenberg, Germany

**Keywords:** hPLEV-F, Extracellular vesicles, Regenerative medicine, Biomaterials, Osteoblasts, Osteoclasts, Bone regeneration, Bone augmentation material, Bioactive material, Human platelet lysate

## Abstract

**Background:**

Bone regenerative medicine focuses on restoring damaged tissue, with bone augmentation materials commonly used to fill defects, support recovery and addressing issues related to aging, bone diseases or trauma in dental and orthopedic procedures. To avoid complications associated with harvesting autogenous tissue grafts, novel applications focus on alloplastic materials to support regenerative and healing processes. However, current synthetic materials demonstrate shortcomings specifically pertaining to mimicking bone regenerative properties of autogenous bone. Whether bioactive fractions enriched for human platelet lysate derived extracellular vesicles (hPLEV-Fs) could biologize alloplastic materials with their non-immunogenic tissue-restorative potential, stimulate intercellular communication between bone-forming osteoblasts and bone-resorbing osteoclasts and transform alloplastic materials in potent regenerative grafts needs to be determined.

**Methods:**

This study investigated hPLEV-Fs impact on bone regenerative pathways and evaluated whether combination with a collagen-embedded β-tricalcium phosphate (β-TCP) three-dimensional matrix enhances bone regeneration.

**Results:**

Treatment with hPLEV-F improved osteoblasts’ proliferation, differentiation and mineralization in both murine and human primary osteoblasts while reducing inflammatory responses, which was further supported by systems-wide phosphoproteome-screening of bone-remodeling pathways. Although initial pre-osteoclastic differentiation was enhanced under hPLEV-F treatment, cells remained in a non-resorbing state, indicating potential for increased net bone formation. Furthermore, hPLEV-F stimulated osteoblasts to increase osteoprotegerin secretion, limiting osteoclast differentiation, especially in combination with β-TCP biomaterial.

**Conclusions:**

Our data demonstrate the potential of hPLEV-F to stimulate bone cell interaction and support bone regenerative pathways, thereby suggesting it as a biologizing agent in combination with synthetic biomaterial. This creates innovative possibilities in biointerface engineering thereby advancing patient care in clinical applications.

**Graphical Abstract:**

①This study examined human platelet lysate-derived extracellular vesicles (hPLEV-F) in bone metabolism. ②hPLEV-F modulated osteoblastic secretion and ③improved differentiation and mineralization in murine and human primary osteoblasts accompanied by enhanced secretion of osteoclastic inhibitor osteoprotegerin (OPG). Moreover, osteoclasts remained in an undifferentiated and non-resorbing state when challenged with hPLEV-F. ④Combination of hPLEV-F and bone augmentation material β-TCPCM enhanced osteoblastic bone regenerative potential and OPG-mediated bone protection, ⑤indicating a role of hPLEV-F in biologizing alloplastic materials for augmentation procedures Created in BioRender. Döding, A. (2025) (https://BioRender.com/xncb1y0).

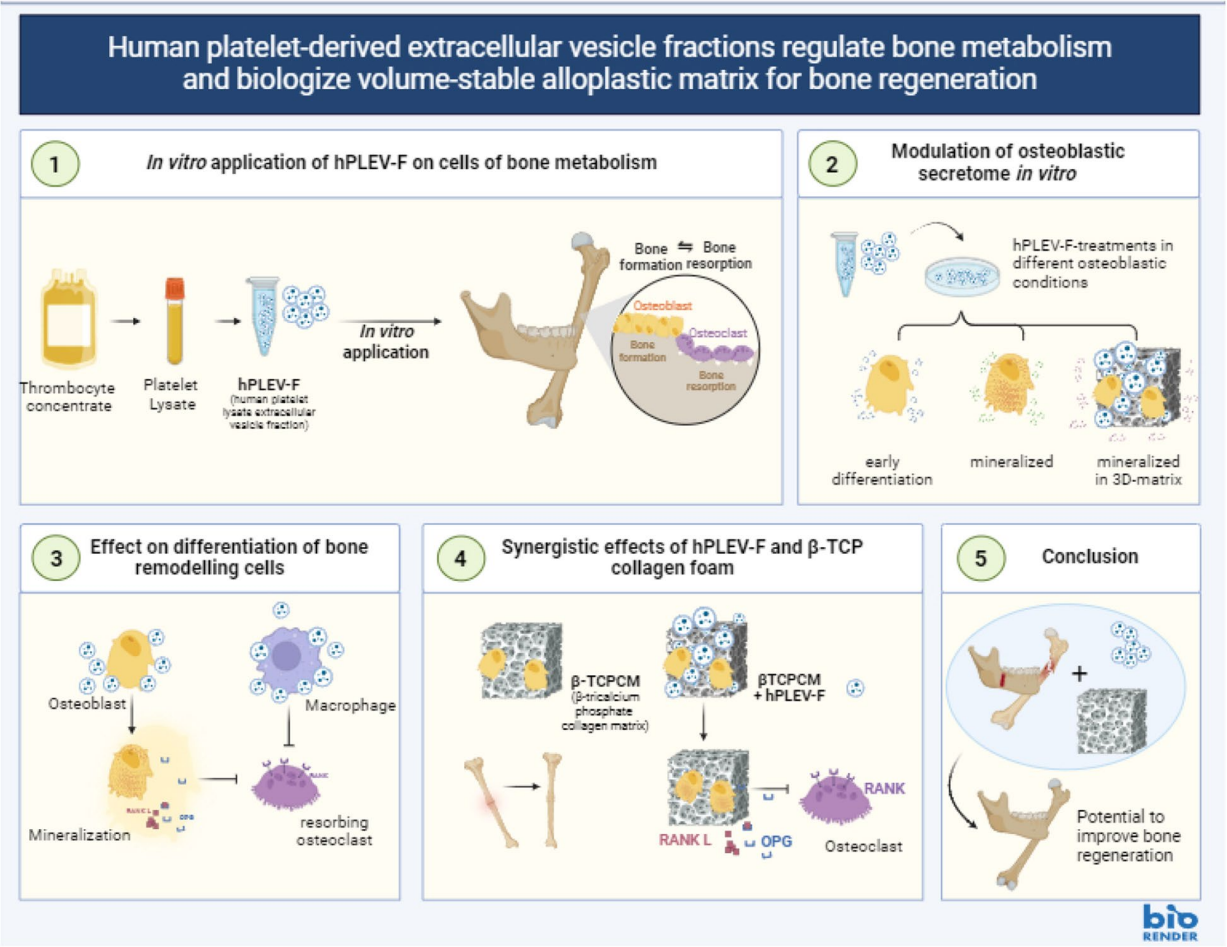

**Supplementary Information:**

The online version contains supplementary material available at 10.1186/s12916-025-04371-w.

## Background

Bone loss is a common sequela associated with aging, various bone metabolic diseases, and trauma [1–3]. In regenerative medicine, bone augmentation materials are often used to fill bony defects and renew damaged bone tissue in its form and function [4]. To avoid drawbacks such as limited availability and donor site morbidity when using autogenous bone, synthetic graft materials are an established alternative [5–7]. However, alloplasts demonstrate shortcomings specifically pertaining to mimicking bone regenerative properties of autogenous bone and current approaches aim to improve material-cell interaction as well as cellular microenvironment [8].

Due to their osteoconductive properties, collagen matrices with embedded β-tricalcium phosphate (β-TCP) are promising alternatives to traditional graft materials [9, 10]. β-TCP degrades during integration and releases calcium and phosphorus during that process, which are both suggested as stimulators of bone formation [11]. However, an intense inflammatory response orchestrated by complex cellular signalling cascades might accompany bone substitute integration [9], targeting bone homeostasis and intervening in bone remodelling. Bone itself is a complex organ whose integrity is influenced by inflammatory cytokines affecting different cell types [12]. Besides osteocytes, fibroblasts and other bone lining cells, bone-forming osteoblasts [13] and bone-resorbing osteoclasts [14] act in concert to maintain a balance between formation and degradation, thereby supporting regeneration. Signalling molecules such as bone morphogenetic proteins (BMPs) and wingless-related integration site pathway (WNT) ligands stimulate osteoblastic differentiation that is characterized by expression of key transcription factors such as runt-related transcription factor-2 (RUNX2), and downstream markers such as alkaline phosphatase (ALP) and collagen type I alpha 1 (COL1A1) [15] contributing to formation of extracellular matrix (ECM) synthesis and mineralization [16, 17]. Moreover, osteoblasts contribute to bone degradation by regulating relevant key factors in maturation of bone resorbing cells [18]. Osteoclast differentiation to multinucleated TRAP-positive cells [19] is stimulated by macrophage colony-stimulating factor (M-CSF) and receptor activator of NF-κB ligand (RANKL), as well as pro-inflammatory cytokine Tumor necrosis factor alpha (TNFα) [20, 21]. Osteoprotegerin (OPG) counteracts the osteoclastogenic effect of RANKL [22]. Since targeted gene expression and specific protein phosphorylation drive cellular signalling processes and thereby integration of bone substitutes, current bone graft research focuses on enhancing osteogenic potential and biological properties by reducing inflammation and improving bone regeneration [4]. In this context, the therapeutic potential of autologous blood-derived biomaterials has been used in various applications, such as wound healing [23], osteoporosis [24, 25], and guided bone regeneration [26–28]. However, preparation of autologous therapeutics mostly requires a complex extraction process at the time of surgery [29]. Hence, there is a growing need for biologized allogeneic materials as adjunct in regenerative procedures.

Novel research approaches are investigating the use of extracellular vesicles (EV), including exosomes and microvesicles, as promising tools in regenerative medicine and other fields [30]. EVs are secreted by various cell types and carry bioactive molecules, such as lipids, nucleic acids, proteins and carbohydrates [31] whose specific composition reflects their originating cells [32] thereby transferring molecular features of their originating cells [33]. In line, EVs impact immune [30] and oncogenic signalling [34] and initial studies described use of exosomes in bone defect repairs in a targeted and non-systemic manner [35]. Of note, these particles possess only low immunogenicity unlike their cells of origin [36]. EV’s isolated from human platelets support angiogenesis [37], neuroprotection [38] or regeneration [39]. Moreover, platelet-derived EVs promoted proliferation, migration and osteogenic differentiation of bone marrow stromal cells (MSCs) resulting in bone-regenerative stimulation in vivo [40, 41]. Variations of preparations for platelet-derived EV’s exist with harvesting protocols ranging from secreted EVs separated from intact platelets, to platelet lysate preparations, in which EVs are enriched by centrifugation, size-exclusion-chromatography and precipitation-methods [35, 37–40]. Exosomes purified from platelet lysates contained elevated levels of key growth factors - vascular endothelial growth factor (VEGF), platelet-derived growth factor-BB (PDGF-BB) and transforming growth factor beta 1 (TGF-β1) - and stimulated bone-forming osteoblasts, identifying them as functional mediators of human platelet lysate [42]. Whether vesicle fractions enriched from platelet lysates ('hPLEV-Fs') could actively promote osteoblasts and their interaction with osteoclasts in inflammatory conditions is not known. Further, their adjunctive use with synthetic biomaterials to improve biological properties and enhance material-cell interaction requires investigation.

Based on hPLEV-F’s suggested properties, we hypothesized that cellular migration within a bone substitution material (β-TCP collagen matrix (β-TCPCM), CERASORB® Foam, Curasan AG) is influenced and bone remodeling capacities of osteoblasts - both in isolation and with regards to their osteoclastogenic capabilities - are impacted. This study investigated for the first time hPLEV-F and its potential to regulate inflammation-induced gene expression and protein secretion of bone-metabolic osteoblasts and osteoclasts in the context of remodelling and regeneration. Furthermore, changes in the osteoblastic phosphoproteome were defined to underline the functional capacity of hPLEV-F.

## Methods

This study investigates the impact of hPLEV-F on bone cell interactions and bone metabolism, as well as its potential in bone regeneration when combined with a β-TCPCM. Based on previous experimental findings [35] and material availability, primary murine cells were used, and key experiments were repeated using human-derived cells to confirm the results. We aimed to characterize the impact of hPLEV-F on bone cell interaction, a prerequisite for bone metabolism, and as an adjunct in bone replacement therapy. Moreover, hPLEV-F was examined in combination with β-TCPCM to assess its potential as a biologizing agent in the context of bone regeneration.

### Characterization and quantification of hPLEV-F

Bioactive fractions enriched for human platelet lysate derived extracellular vesicles (hPLEV-Fs) were commercially obtained from Lysatpharma GmbH (Eisenberg, Germany) (Germany/European Patent: EP3468568). The hPLEV-Fraction was fabricated using thrombocyte concentrates according to disclosed internal company protocols for enrichment of EV. As a source material, surplus apheresis concentrates of minimum 10 different healthy donors were purchased from a blood bank (Institut für Klinische Transfusionsmedizin Jena gGmbH (IKTJ)), pooled and used for further processing. The hPLEV-Fraction was qualified according to recommended ISEV-guidelines [43]. Western Blot, nanoparticle-tracking analysis (NTA) and electron microscopy was performed by the manufacturer (data available upon request). For confirmation of results with different batches, we used hPLEV-F Batch 2 (#01/22) and hPLEV-F Batch 3 (#10/22). Mode values captured by nanoparticle tracking analysis 145 nm after one freezing/thawing cycle according to the manufacturer.

### Murine osteoblast experiments

#### Material for primary cell cultures

**Fig. 1:**
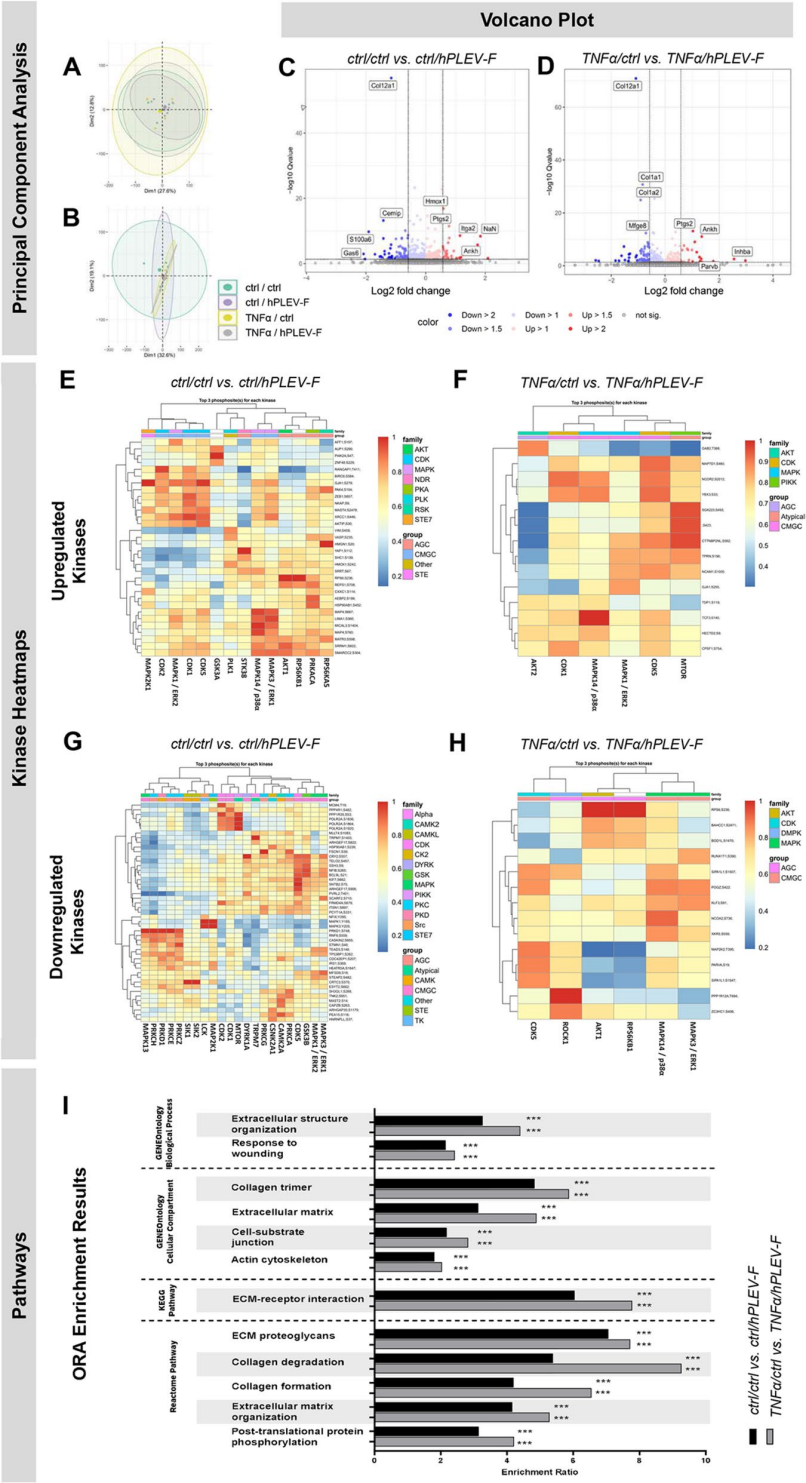
Treatment with hPLEV-F leads to selectively altered phosphorylation patterns in osteoblasts. Phosphoproteomic analysis of primary murine osteoblasts after treatment with human platelet lysate-derived extracellular vesicle fraction (hPLEV-F) (14 h) and/or TNFα (30 min) (ctrl/hPLEV-F, TNFα/hPLEV-F, TNFα/ctrl) in comparison to untreated cells (ctrl/ctrl). **A**,** B** Principal component analysis (PCA) for (**A**) whole cell and (**B**) phosphoenrichment analysis. **C**,** D** Volcano plots depicted as log2 fold change of protein expression plotted against log10 *p* value of each feature. Comparison of hPLEV-F prestimulated osteoblasts (**C**) without (ctrl/ctrl vs ctrl/hPLEV-F) and (**D**) with additional TNFα-stimulus (TNFα/ctrl vs TNFα/hPLEV-F) in comparison to respective control. **E**–**H** Heatmap depiction of predicted up- (**E**, **F**) and downregulated (**G**, **H**) kinases under hPLEV-F-treatment without (**E**,** G**) and with (**F**,** H**) inflammatory TNFα-stimulus. **I** Bone remodeling pathways derived from over representation analysis (ORA) hPLEV-F treatment (black bars) and TNFα and hPLEV-F treatment (grey bars) depicted as enrichment ratio of hPLEV-F-untreated respective control. All data for the phosphoproteome analysis are based on four different biological replicates of each monitored condition. *hPLEV*-*F* human platelet lysate-derived extracellular vesicle fraction, *ORA* over representation analysis, *ctrl* untreated control. ***p < 0.001

**Fig. 2:**
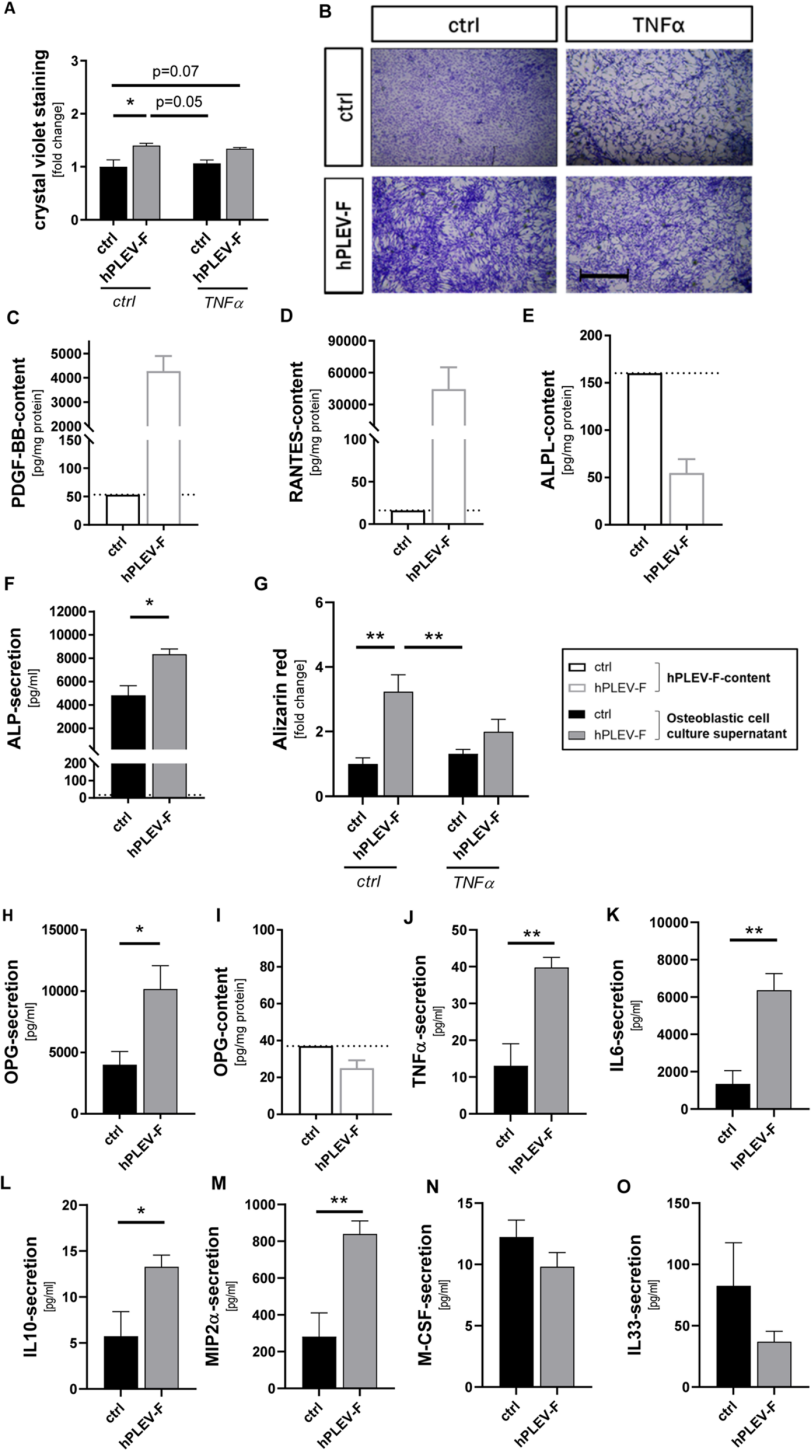
Treatment with hPLEV-F ameliorates osteoblastic proliferation and differentiation. **A**, **B** Murine osteoblast (mOB) proliferation activity was analysed in primary cultures after 3 days of proliferation using crystal violet staining. Cells were treated with hPLEV-Fs with and without inflammatory trigger (TNFα). **A** Photometric quantification of crystal violet is depicted as fold change of untreated cells (ctrl/ctrl). **B** Crystal violet stained mOBs under the different conditions (ctrl/ctrl, ctrl/hPLEV-F, TNFα/ctrl and TNFα/hPLEV-F). **C**–**E** The hPLEV-F-lysates were analysed for content of PDGF-BB (**C**), RANTES (**D**) and ALPL (**E**) in comparison to a control (EV-F of pooled human plasma). Differentiation of mOB was analysed in quantitative ALP-ELISA (**F**) from supernatant after 7 days of differentiation and after 14 days quantified by alizarin red staining (**G**). The photometric quantification of alizarin red assay is given as fold change of respective control (ctrl/ctrl). ELISA-measurements of osteoblastic OPG-secretion (**H**) and averaged OPG within the hPLEV-Fs (**I**) was performed. **J**–**O** The hPLEV-F-dependent changes in secretion of TNFα (**J**), IL-6 (**K**), IL-10 (**L**), MIP2α (**M**), M-CSF (**N**) and IL-33 (**O**) from 7-days-predifferentiated mOB. Dotted lines in (**C**-**E**, **I**) indicating level of control. Dotted line in (**F**) indicating level of added ALPL by hPLEV-F: 18 pg/ml; *hPLEV*-*F* human platelet lysate-derived extracellular vesicle fraction; Statistical analyses: **C**, **D**, **E**, **I**: no statistical analyses, n of 1 for control; **F**, **H**, **J**–**O**: Student’s *t* test **A**, **G** two-way ANOVA with Tukey´s post hoc tests; *p < 0.05; **p ≤ 0.01 Scale bar in (**B**) 1 mm. Experiments were performed in at least three independent biological replicates. Legend describes coloration of bars in combination with underling experimental setup in Fig. 2

**Fig. 3:**
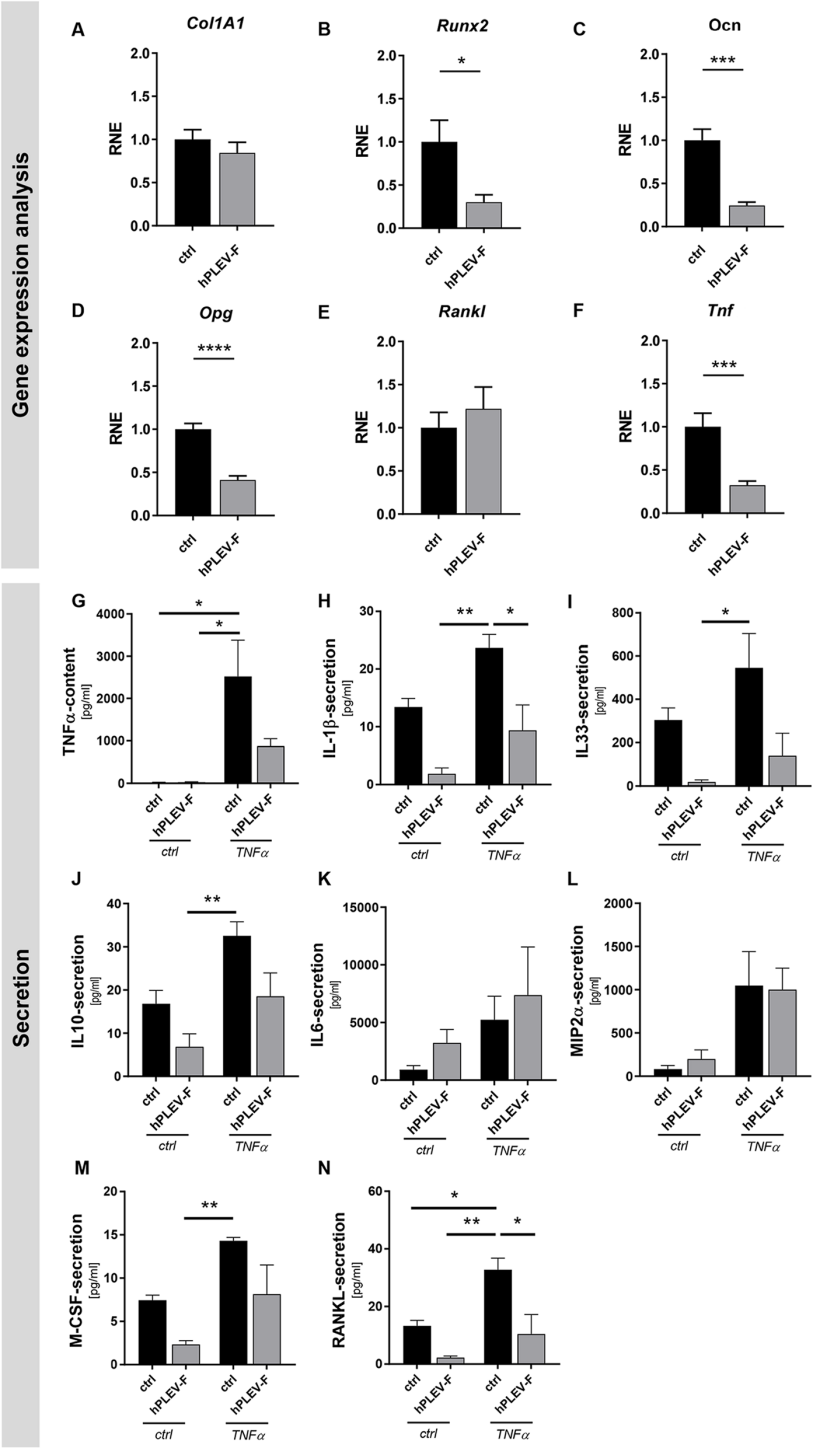
hPLEV-treatment reduces TNFα and cytokine secretion specifically in inflammatory conditions. After 5 days of differentiation, primary murine osteoblasts were treated with hPLEV-F. **A**–**F** Gene expression levels of **A** *Col1A1*, **B** *Runx2*, **C** *Ocn*, **D** *Opg*, **E** *Rankl* and **F** *Tnf* are depicted as fold change of ctrl cells. **G**–**N** Following hPLEV-F-pretreatment, TNFα-stimulus was applied for 24 h. ELISA-based quantification of hPLEV- and TNFα-challenged secretions of (**G**) IL-1β (**H**), IL-33 (**I**), IL-10 (**J**), IL-6 (**K**), MIP2α (**L**), M-CSF (**M**) and RANKL (**N**) are shown in pg/ml. *hPLEV*-*F* human platelet lysate-derived extracellular vesicle fraction. Statistical analyses: **A**–**F** Student’s *t* test. **G-N** two-way ANOVA with Tukey’s post hoc tests; *p < 0.05; **p ≤ 0.01; ***p ≤ 0.001; ****p ≤ 0.0001. Experiments were performed in at least three independent biological replicates

**Fig. 4:**
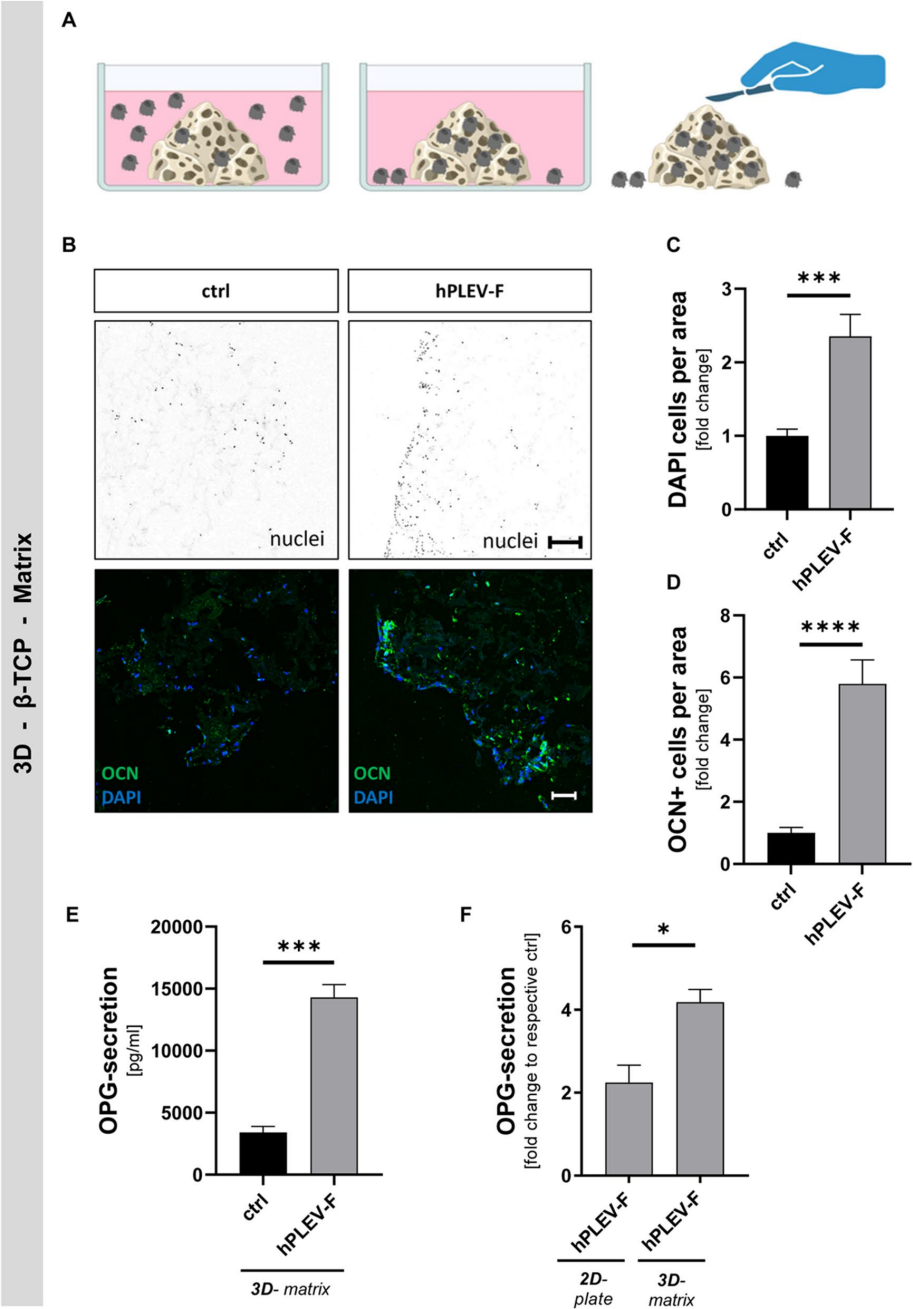
hPLEV-F-treatment improves differentiation of matrix-seeded cells and enhances bone-protecting properties. **A** mOB were seeded in a three-dimensional resorbable ceramic-collagen foam matrix with incorporated β-tricalcium phosphate granules (β-TCPCM (CERASORB® Mouldable Foam, Curasan AG)) and cultivated with differentiation medium for 15 days. **B** Following fixation and slide preparation, cells were stained for Osteocalcin (OCN) (green) and DAPI (blue). The black and white pictures were prepared to depict only the DAPI staining. **C** Microscopic pictures were quantified for cellular abundance per area (DAPI cell core staining) and given as fold change of control cells (ctrl). **D** OCN^+^ cells were quantified and given as fold change of control cells (ctrl). **E**, **F** Cell culture supernatant was analysed for OPG-secretion (**E**) from cells cultured in β-TCPCM (‘3D’) in pg/ml and (**F**) as fold change of respective control in classical cell culture conditions (‘2D’) compared to 3D on β-TCPCM. *β*-*TCP* β-tricalcium phosphate, *OCN* osteocalcin, *hPLEV*-*F* human platelet lysate-derived extracellular vesicle fraction. Statistical analyses: Student’s *t* test; *p < 0.05; ***p ≤ 0.001; ****p ≤ 0.0001; experiments were performed in at least three independent biological replicates. Scale bar in (B-black/white) 200 µm and (B-blue/green) 50 µm. Graphic in (**A**) was created with BioRender, Döding, A. (2025) (https://BioRender.com/9d6hn8q)

**Fig. 5:**
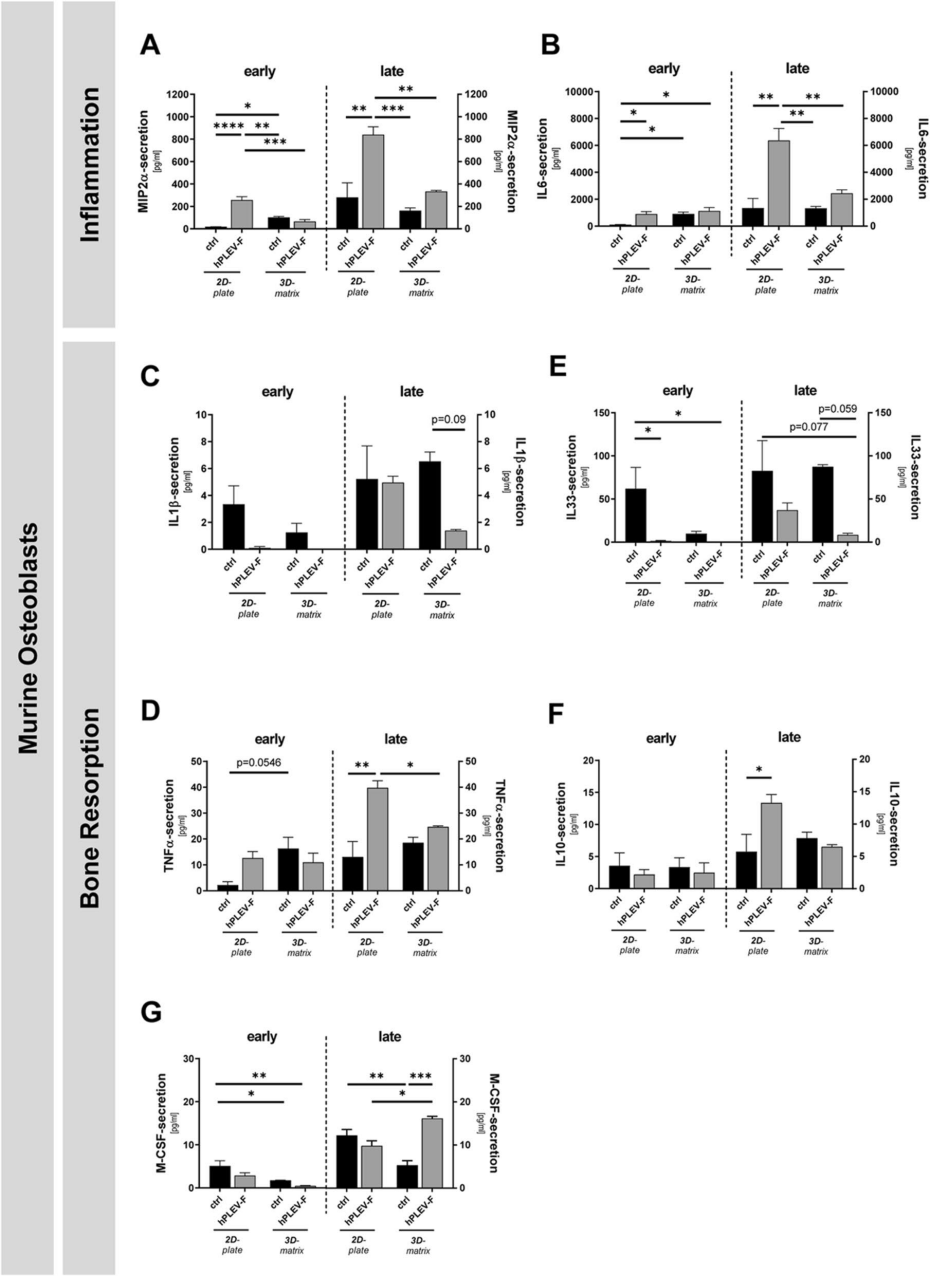
Treatment with hPLEV-F balances pro- and anti-inflammatory signalling. Changes in differentiation and treatment-dependent (**A**, **B**) inflammatory and (**C**–**G**) bone remodelling cytokines were measured in Multiplex immunoassay. Changes in secretion at the early (4/5 days) and late (14 days) phase and between 2D- and 3D-cultured murine osteoblasts were investigated in presence and absence of human platelet lysate-derived extracellular vesicle fraction (hPLEV-F). Secretion levels of **A** MIP2α, **B** IL-6, **C** IL-1β, **D** TNFα, **E** IL-33, **F** IL-10 and **G** M-CSF were analysed and depicted in pg/ml. *hPLEV*-*F* human platelet lysate-derived extracellular vesicle fraction. Experiments were performed in at least three independent biological replicates. Statistical analyses: two-way ANOVA with Tukey’s post hoc tests. *p < 0.05; **p ≤ 0.01; ***p ≤ 0.001; ****p ≤ 0.0001

**Fig. 6:**
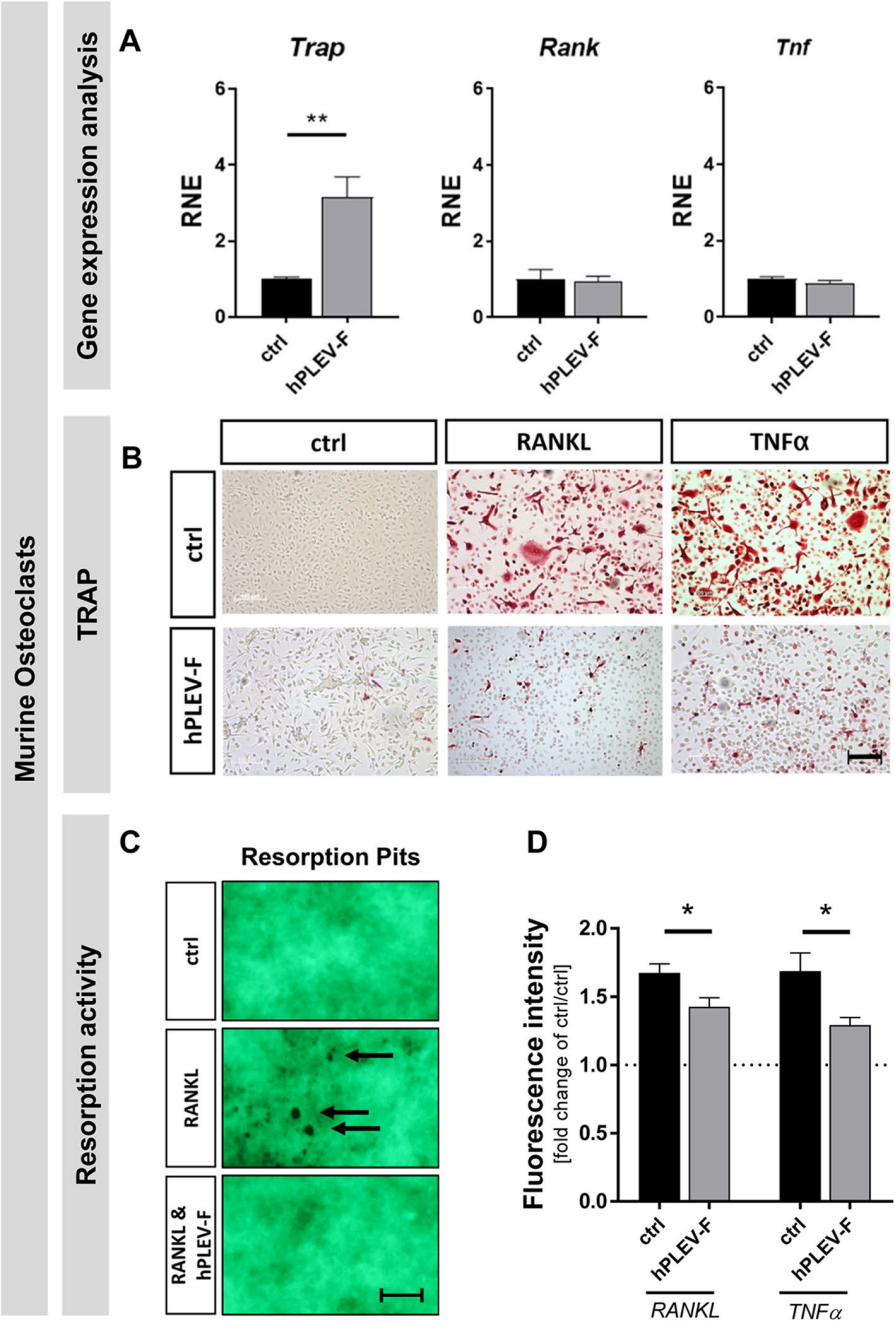
hPLEV-F reduces TNFα- and RANKL-mediated osteoclast differentiation and resorption activity. Murine bone marrow cells (BMC) were stimulated (**A**) with M-CSF or (**B**) with M-CSF and RANKL supplemented medium in presence and absence of hPLEV-F. **A** After 48 h of treatment in absence (ctrl) or presence of human platelet lysate-derived extracellular vesicle fraction (hPLEV-F), cells were investigated for *Trap*-, *Rank*- and *Tnfα*-expression depicted as fold change of unstimulated cells (ctrl). **B** Cells were stained for TRAP after 5 days of TNFα/RANKL-triggered differentiation under hPLEV-F. **C**, **D** For quantification of resorption activity, BMC were seeded on a calcium phosphate/fluoresceinamine-labeled chondroitin sulphate (FACS) coated plate and differentiated under M-CSF/RANKL- or -/TNFα-conditions with or without hPLEV-F-treatment. **C** Resorption pits became visible on the bottom of the dish (indicated by black arrows in **C**). **D** Release of the FACS into supernatants was quantified and depicted as fold change of unstimulated control (dashed line in **D**). *BMC* bone marrow cells, *hPLEV*-*F* human platelet lysate-derived extracellular vesicle fraction, *FACS* fluoresceinamine-labeled chondroitin sulphate. *hPLEV*-*F* human platelet lysate-derived extracellular vesicle fraction. Experiments were performed in at least three independent biological replicates. Statistical analyses: Student’s *t* test; **p ≤ 0.01 (in **A**); *p < 0.05 (compared to respective control) (**D**). Scale in (**B**) 100 µm and (**C**) 150 µm

Murine primary cellular material was isolated from long bones of ≥ 4-week-old male C57Bl6 mice. Mice were bred in-house (animal facility of the Jena University Hospital) and housed under standardized laboratory conditions. Mice were kept in individually ventilated cages according to institutional guidelines and received food and water ad libitum. Animal husbandry was carried out in accordance with the Council Directive of the European Community for the Care and Use of Laboratory Animals (2010/63/EU) and German legislation. Following German animal welfare law, organ removal without pre-treatment of the animals is subject to an internal license: twz21-2017. The data sets generated using primary murine cells are shown in Figs. 1, 2, 3, 4, 5 and 6.

#### Isolation and cultivation of osteoblast cultures

Primary osteoblasts were isolated from long bones of the fore and hind limbs as previously described [44]. Briefly, after removal of soft tissue and centrifuging out the bone marrow [45], bones were washed in Dulbecco’s phosphate-buffered saline (DPBS, GibcoTM, Thermo Fisher Scientific, pH 7.0–7.3) and cut into small pieces prior to collagenase digestion (500 U/ml Collagenase II, Worthington) in Dulbecco’s modified Eagle’s medium low glucose (DMEM low glucose, pH 7.0–7.4, GibcoTM, Thermo Fisher Scientific), for 2 h at 37 °C. Bone fragments were washed three times in proliferation medium (DMEM low glucose, 100 U/ml penicillin, 100 µg/ml streptomycin (Thermo-Fisher Scientific), 50 µg/ml gentamycin (Sigma-Aldrich) and 1.25 µg/ml Fungizone (Sigma-Aldrich)) and transferred to a T25-flask with 5 ml proliferation medium. The medium was changed for the first time after 5 days of incubation. Subsequent media changes were performed three times per week until cells reached confluency. Cells were subjected to passaging and cultivated until they reached purity (~ P3). They were expanded and subsequently used in the described experiments.

#### Cell culture treatment with hPLEV-F

hPLEV-F are resolved in 0.9% NaCl-solution in a concentration of 3.75 mg/ml. In all cell culture experiments, an effective concentration of 187.5 µg hPLEV-F/ml (5% of final volume) medium was used.

#### Osteoblastic pretreatment for phosphoproteomic analysis

For the phosphoproteomic analysis, murine osteoblasts were seeded at 1.0 × 10^5^ in a 6-well plate in proliferation medium (DMEM low glucose, 100 U/ml penicillin, 100 µg/ml streptomycin (Thermo-Fisher Scientific), 50 µg/ml gentamycin (Sigma-Aldrich) and 1.25 µg/ml Fungizone (Sigma-Aldrich)). After 48 h of incubation at 37 °C, proliferation medium was renewed and hPLEV-F (Lysatpharma) were added. After another 24 h, the TNFα stimulus was applied 30 min prior to ending the experiment. Cells were then washed twice with DPBS supplemented with 1% protease/phosphatase inhibitor (Halt™ Protease Inhibitor Cocktail, Thermo Fisher Scientific) + 1% EDTA) on ice and then detached with a cell scraper (Sarstedt) from the bottom of the well in 500 µl washing buffer. The suspension was then centrifuged at 1000 × g for 10 min at 4 °C. Supernatant was removed and the pellet frozen at − 80 °C.

#### Protein isolation for phosphoproteomic analysis

After thawing, lysis buffer was added to the samples to a final concentration of 5% sodium dodecyl sulfate (SDS), 100 mM 4-(2-hydroxyethyl)−1-piperazineethanesulfonic acid (HEPES) and 50 mM dithiothreitol (DTT). The samples were sonicated (Bioruptor Plus) for 10 cycles (30 s ON/60 s OFF) at a high setting at 20 °C, followed by boiling at 95 °C for 7 min. Reduction was followed by alkylation with iodoacetamide (final concentration 15 mM) for 30 min at room temperature in the dark. Samples were acidified with phosphoric acid (final concentration 2.5%), and seven times the sample volume of S-trap binding buffer was added (100 mM triethylammonium bicarbonate (TEAB), 90% methanol). Samples were bound on 96-well S-trap micro plate (Protifi) and washed three times with binding buffer. Trypsin in 50 mM TEAB pH 8.5 was added to the samples (1 µg per sample) and incubated for 1 h at 47 °C. The samples were eluted in three steps with 50 mM TEAB pH 8.5, elution buffer 1 (0.2% formic acid in water) and elution buffer 2 (50% acetonitrile and 0.2% formic acid). Before phosphopeptide enrichment, samples were filled up to 210 µl. Phosphorylated peptides were enriched using Fe(III)-IMAC cartridges (Agilent) in an automated fashion using the standard protocol from the AssayMAP Bravo Platform (Agilent Technologies). In short, Fe(III)-IMAC cartridges were first primed with 100 µl of priming buffer (0.1% Trifluoroacetic acid (TFA) in pure Acetonitrile (ACN)) and equilibrated with 50 μl of OASIS elution buffer. After loading the samples into the cartridge, the cartridges were washed with OASIS elution buffer, while the syringes were washed with priming buffer. The phosphopeptides were eluted with 25 μl of 1% ammonia directly into 25 μl of 10% formic acid (FA). Samples were dried down with a speed vacuum centrifuge and stored at − 20 °C until liquid chromatography–mass spectrometry (LC–MS) analysis. For whole proteome analysis, the flow through after phosphoenrichment was dried down with a speed vacuum centrifuge and stored at − 20 °C until LC–MS analysis.

#### Liquid chromatography–mass spectrometry and data independent analysis

For whole cell proteomics on Evosep, samples were loaded on Evotips (Evosep) according to the manufacturer’s instructions. In short, Evotips were first washed with Evosep buffer B (acetonitrile, 0.1% formic acid), conditioned with 100% isopropanol and equilibrated with Evosep buffer A. Afterwards, the samples were loaded on the Evotips and washed with Evosep buffer A. The loaded Evotips were topped up with buffer A and placed in the machine. Peptides were separated using the Evosep One system (Evosep) equipped with a 15 cm × 150 μm i.d. packed with a 1.5 μm Reprosil-Pur C18 bead column (Evosep Performance, EV-1137, PepSep) heated at 45 °C with a butterfly sleeve oven (Phoenix S&T). The samples were run with a pre-programmed proprietary Evosep gradient of 44 min (30 samples per day) using water and 0.1% formic acid and solvent B acetonitrile and 0.1% formic acid as solvents. The liquid chromatography (LC) was coupled to an Orbitrap Exploris 480 (Thermo Fisher Scientific) using PepSep Sprayers and a Proxeon nanospray source. The peptides were introduced into the mass spectrometer via a PepSep Emitter 150 μm outer diameter × 10 μm inner diameter, heated at 300 °C and a spray voltage of 2 kV was applied. The injection capillary temperature was set at 300 °C. The radio frequency ion funnel was set to 30%. For data-independent acquisition (DIA), full scan mass spectrometry (MS) spectra with a mass range of 350–1650 m/z were acquired in profile mode in the Orbitrap with a resolution of 120,000 full width at half maximum (FWHM). The default charge state was set to 2 +, and the filling time was set at a maximum of 20 ms with a Limitation of 3× 10^6^ ions. DIA scans were acquired with 40 mass window segments of differing widths across the MS1 mass range. Higher collisional dissociation fragmentation (normalized collision energy 30%) was applied, and MS/MS spectra were acquired with a resolution of 30,000 FWHM with a fixed first mass of 200 m/z after accumulation of 1 × 10^6^ ions or after filling time of 45 ms (whichever occurred first). Data were acquired in profile mode. For data acquisition and processing of the raw data, Xcalibur 4.4 (Thermo) and Tune version 4.0 were used.

For phosphopeptides, samples were reconstituted in in MS Buffer (5% acetonitrile, 95% Milli-Q water, with 0.1% formic acid) and spiked with iRT peptides (Biognosys). Peptides were separated in trap/elute mode using the nanoAcquity ultra-high performance liquid chromatography system (Waters, Waters Corporation) equipped with a trapping (Waters nanoEase M/Z Symmetry C18, 5 μm, 180 μm × 20 mm) and an analytical column (Waters nanoEase M/Z C18 HSS T3, 1.7 μm, 75 μm × 250 mm). Solvent A was water and 0.1% formic acid, and solvent B was acetonitrile and 0.1% formic acid. Then, 5 µl of the sample were loaded with a constant flow of solvent A at 5 μl/min onto the trapping column. Trapping time was 6 min. Peptides were eluted via the analytical column with a constant flow of 0.3 μl/min. During the elution step, the percentage of solvent B increased in a nonlinear fashion from 0 to 40% in 60 min. Total run time was 75 min. The LC was coupled to an Orbitrap Fusion Lumos (Thermo Fisher Scientific) using the Proxeon nanospray source. The peptides were introduced into the mass spectrometer via a Pepsep emitter with integrated Liquid Junction 150 μm outer diameter × 20 μm inner diameter, 2.6 cm length (Bruker) heated at 300 °C, and a spray voltage of 2 kV was applied. The capillary temperature was set at 300 °C. The radio frequency ion funnel was set to 30%. Full scan mass spectrometry (MS) spectra with mass range 350–1650 m/z were acquired in profile mode in the Orbitrap with resolution of 120,000 FWHM. The default charge state was set to 3 +. The filling time was set at maximum of 60 ms with Limitation of 3× 10^6^ ions. DIA scans were acquired with 40 mass window segments of differing widths across the MS1 mass range. Higher collisional dissociation fragmentation (stepped normalized collision energy; 25, 27.5 and 30%) was applied and MS/MS spectra were acquired with a resolution of 30,000 FWHM with a fixed first mass of 200 m/z after accumulation of 3 × 10^6^ ions or after filling time of 35 ms (whichever occurred first). Data were acquired in profile mode. For data acquisition and processing of the raw data, Xcalibur 4.5 (Thermo) and Tune version 4.0 were used.

#### Data analysis

DIA raw data were analysed using the directDIA pipeline in Spectronaut (v.18, Biognosysis). The data were searched against a species specific (Mus Musculus, 16.747 entries) and a contaminants (247 entries) Swissprot database.

For whole proteome analysis, the data were searched with the following variable modifications: Oxidation (M) and Acetyl (Protein N-term). A maximum of 2 missed cleavages for trypsin and 5 variable modifications were allowed. The identifications were filtered to satisfy FDR of 1% on peptide and protein level. Relative quantification was performed in Spectronaut for each paired comparison using the replicate samples from each condition. The data (candidate table) and data reports (protein quantities) were then exported and further data analyses and visualization were performed with Rstudio using in-house pipelines and scripts. To select significant proteins, a logarithmic foldchange (log2FC) cutoff of 0.58 and a *q* value < 0.05 were defined.

For phosphoproteomic analysis, the data were searched with the following modifications: Carbamidomethyl (C) (Fixed) and Oxidation (M), Acetyl (Protein N-term), Phospho (STY) (Variable). Post-translational modification (PTM) localization probability was set to 0.75 and consolidation of phosphosites was sum based. A maximum of 2 missed cleavages for trypsin and 5 variable modifications were allowed. The identifications were filtered to satisfy false discovery rate (FDR) of 1% on peptide and protein level. Relative quantification at phosphosite level was performed in Spectronaut for each paired comparison using the replicate samples from each condition. The data (candidate table) and data reports (protein quantities) were then exported and further data analyses and visualization were performed with Rstudio using in-house pipelines and scripts. To select significant phosphosites, a log2FC cut-off of 0.58 and a *q* value < 0.05 were defined. For identifying the most regulated kinases and the corresponding regulated top phosphosites, a modified version of PhosR was used. Signalling pathways were analysed by the over-representation analysis (ORA) method using the WEB-based GEne SeT AnaLysis Toolkit available at http://www.webgestalt.org/ [46], with GENEOntology Biological Process, GENEOntology Cellular Compartment, KEGG and Reactome as functional databases. Data are based on four different biological replicates of each monitored condition.

#### Osteoblast proliferation activity

Osteoblast proliferation activity was determined using a crystal violet assay (Sigma-Aldrich) as previously described [47]. Briefly, cells were seeded in 24-well plates at a concentration of 1.4 × 10^4^ cells/well in proliferation medium (composition mentioned above). TNFα, a pro-inflammatory cytokine elevated in various bone pathologies such as periodontitis, rheumatoid arthritis, osteomyelitis and fracture healing was used to mimic a pro-inflammatory milieu [48]. Briefly, after being in culture for 24 h, hPLEV-F (Lysatpharma) were added, before including 50 ng/ml TNFα (PeproTech) as pro-inflammatory stimulus for additional 12 h of incubation. After 3 days, cells were washed with PBS (pH 7.0–7.4) and stained using 100 µl of a crystal violet solution (0.5% (w/v) crystal-violet-powder, 20% methanol in ddH_2_O) for 15 min. Cells were washed three times with ddH_2_O, cells and photographed using a camera (EOS 77D, Canon), mounted on an inversed microscope (Primovert iLED Microscope, Carl Zeiss). Afterwards, plates were dried for 4 h at 37 °C until the dye was dissolved using 120 µl/well methanol. Samples were measured using a photometric Infinite M Nano plate reader (Tecan Life Science) at an absorption of 595 nm. Data are based on four different biological replicates of each monitored condition. Each sample was analysed in duplicates in a 48-well plate. Measured crystal violet intensities were depicted as fold change of control group.

#### Osteoblast mineralization activity/Alizarin red staining

Cells were seeded in a density of 2.8 × 10^4^ cells per well on 24-well plates (Day 0). After 2 days in culture, cells reached approximately 80% confluency and the proliferation medium was exchanged for differentiation medium (proliferation medium supplemented with 100 µg/ml l-ascorbic acid, 10 mM β-glycerol phosphate and 100 nM dexamethasone). At the same time hPLEV-F (Lysatpharma) was added, and a TNFα-trigger (50 ng/ml TNFα (PeproTech)) was applied (Day 3). Media change took place after every 72-h period with freshly adding hPLEV-Fs and TNFα (Day 4, 7, 10 and 13 after change to differentiation medium). Supernatants for secretion analysis were collected on days 5 and 14. Osteoblast mineralization activity was determined using an alizarin red assay (Sigma-Aldrich) according to the manufacturer’s recommendations. Briefly, on day 15 after change to differentiation medium, cells were fixed for 15 min in 10% paraformaldehyde (PFA) and incubated with 40 mM alizarin red solution (in ddH_2_O; pH 4.1–4.3) for 20 min. Free alizarin red was washed away with water (ddH_2_O) prior to cell lysis using 10% acetic acid for 30 min. Afterwards, the suspension was heated to 85 °C for 10 min and allowed to cool down for 5 min on ice. Then, samples were centrifuged for 15 min at 20,000 × g and the pH of the supernatant was neutralized with 10% ammonium hydroxide. Values were determined twice for each well at 405 nm (Infinite M Nano plate reader, Tecan Life Science). Concentrations were determined by comparing values to a standard curve and normalized to an untreated control. The experiment was performed with three biological replicates per condition. Each biological replicate had two technical replicates. Wells were photographed using a camera (EOS 77D, Canon) mounted on an inversed microscope (Primovert iLED Microscope, Carl Zeiss).

#### Osteoblast gene expression and secretome

Cells were seeded in a density of 2.8 × 10^4^ cells per well on 24-well plates. After 2 days in culture, cells reached approximately 80% confluency and the proliferation medium was exchanged for differentiation medium (proliferation medium supplemented with 100 µg/ml l-ascorbic acid, 10 mM β-glycerol phosphate and 100 nM dexamethasone). Cells were allowed to differentiate over 6 days prior to hPLEV-F-application. After 24 h hPLEV-F-pre-treatment (Lysatpharma), a TNFα-trigger (50 ng/ml TNFα (PeproTech)) was applied for further 24 h. Supernatant was collected for enzyme-linked immunosorbent assay (ELISA) and Multiplex Immunoassay prior to cell lysis with TRIzol for gene expression analyses. For gene expression analysis, experiments were performed in three independent biological replicates. Gene expression analysis was performed according to ‘Gene expression analysis’ section and secretome analysis via Multiplex as described in ‘Multiplex immunoassay’ section.

#### Immunofluorescence staining of osteoblasts

Osteoblasts were seeded in a concentration of 1.4 × 10^4^ cells on rat-tail collagen Type I (Sigma-Aldrich)-coated coverslips in proliferation medium. After 72 h, when cells reached 80% confluency medium was exchanged for differentiation medium (described above) and hPLEV-F-treatment (Lysatpharma) started. After 14 days of treatment with hPLEV-Fs, with medium change every 2–3 days, cells were fixed in 4% PFA for 5 min, washed with PBS (pH 7.4) three times, prior to preconditioning using 1.5% normal-goat serum (NGS) + 0.1% TX-100 (Triton). Then the anti-osteocalcin-antibody (rabbit ab93876, Abcam, 1:500 in 1.5% NGS in PBS) was applied overnight at 4 °C. After 3 washing cycles using PBS, the secondary antibody was applied (Alexa Fluor 488 anti-rabbit IgG (H + L), Invitrogen, 1:1,000 in 1.5% NGS). Thereafter, nuclei were stained with DAPI (Thermo Fisher Scientific; 1:10,000 in PBS). After washing the excess dye using three times PBS and one time ddH_2_O, coverslips were mounted on slides using Mowiol (Mowiol 4–88, Carl Roth). Experiments were performed in three independent biological replicates. Imaging was performed with the inverted confocal laser scanning microscope TCS SP5 (Leica).

#### Bone augmentation matrix

Osteoblast were cultured on a resorbable ceramic-collagen foam with incorporated β-tricalcium phosphate (β-TCP) granules (CERASORB® Mouldable Foam, Curasan AG). It is composed of 85% β-TCP particles, ranging from 150 to 2000 μm in diameter, and 15% porcine collagen, including 80% type I collagen, 15% elastin and 5% type III collagen [49]. The granules have a porosity of 65% and a pore size of 0.1–500 µm [50]. The foam dimensions were approximately 0.6 cm × 0.6 cm × 0.2 cm cubes and cells were seeded in a density of 1.4 × 10^4^ cells/well in a 24-well plate with 600 µl proliferation medium (as described above), covering the foam completely. After 2 days, the medium was exchanged for a differentiation medium as described above. At the same time, hPLEV-F (Lysatpharma) was added, and a TNFα-trigger (50 ng/ml TNFα (PeproTech)) was applied. Then, 0.9% NaCl was used as a control condition to hPLEV-F-treatment. On day 4, 7, 10 and 13 after the change to differentiation media medium was changed. On day 4 and 14 of differentiation, supernatants were collected for analysis of protein secretion. On day 15 of differentiation, samples were fixed with 4% PFA for 15 min and washed for 2 cycles with PBS prior to paraffin-embedding. This experiment was performed with four biological replicates and two technical replicates.

#### Immunofluorescence staining of osteoblasts within the bone substitute matrix

Fixed samples were processed overnight in a Histokinette (Tissue processor TP1020, Leica) and embedded (Histocore Arcadia modular tissue embedding system, Leica) in paraffin (Histosec® Pastilles, Merck). Samples were cut in 9-µm-thick slices using a Microtome (Rotary Microtome RM2265, Leica).

For cellular osteocalcin staining, slides were deparaffinised in an increasing ethanol series and washed twice with PBS (pH 7.4). To reduce background, slices were treated with 1.5% normal-goat serum (NGS) + 0.1% TX-100 (Triton) in PBS. Slices were stained with primary anti-osteocalcin-antibody (rabbit ab93876, Abcam, 1:500 in 1.5% NGS) overnight at 4 °C. Visualization was performed with secondary antibody was applied (Alexa Fluor 488 anti-rabbit IgG (H + L), Invitrogen, 1:1,000 in 1.5% NGS). Nuclei were counterstained using a 4′,6-diamidino-2-phenylindole, dihydrochloride (DAPI) core staining (Thermo Fisher Scientific) 1:10,000 in PBS. Slices were embedded with Mowiol (Mowiol 4–88, Carl Roth). All four biological replicates and their two technical replicates of each condition has been stained and analysed.

#### Evaluation of osteocalcin immunofluorescence

Imaging for the evaluation of osteocalcin activation and immunofluorescence staining was performed with an inverted confocal laser scanning microscope TCS SP5 (Leica). Slides were scanned in multiple layers and then organized in z-stacks. To analyse the number of DAPI-positive and osteocalcin cells, the Fiji software (https://imagej.net/Fiji; version number 1.54g, accessed on 26 January 2024) was used. DAPI cells were automatically counted in the entire slides and randomly checked visually. Osteocalcin-positive cells were visually counted in four randomly selected (two with edge, two without edge) equally sized sections for each cut. Microscopic imaging of each experiment was performed under identical settings for each treatment condition (ctrl and hPLEV-F-treated). All four biological replicates and their two technical replicates of each condition have been stained and analysed.

### Murine osteoclast experiments

#### Osteoclast cultures

As described previously [45], murine bone marrow cells (BMC) were centrifuged out of the long bones of the front and hind limbs (10,000 × g for 20 s) of C57BL6 mice. BMC were resuspended in osteoclast proliferation medium (αMEM containing 10% FBS (Gibco) and 1% penicillin/streptomycin) and incubated overnight in 10 ml proliferation medium on a 10 cm dish (one per animal). For treatment with hPLEV-F, non-adherent cells were seeded in a density of 3 × 10^5^ cells per well in 600 µl proliferation medium 50 ng/ml macrophage colony-stimulating factor (M-CSF) (PeproTech) in 24-well plates on round coverslips (Menzel™Microscopic Coverslips round 8 mm, Thermo Fisher Scientific). Murine osteoclast cultures were used for the experiments depicted in Fig. 6.

#### Pre-osteoclastic differentiation

Following the overnight incubation on a 10 cm dish, non-adherent cells were seeded in a density of 0.3 × 10^6^ cells per 24-well in a total volume of 0.6 ml differentiation medium (proliferation medium supplemented with 50 ng/ml M-CSF). The following day, 80% of medium were changed and cells were supplemented with 187.5 µg/ml hPLEV-F (Lysatpharma). After 24 h, 50 ng/ml TNFα (PeproTech) or 20 ng/ml RANKL (PeproTech) were added for further 24 h, before cells were lysed with TRIzol and RNA-expression was quantified. Gene expression analysis is described in ‘Gene expression analysis’ section.

#### Osteoclast differentiation

Osteoclast differentiation was examined using a tartrate-resistant acid phosphatase (TRAP) staining. Selected wells were treated with hPLEV-F (Lysatpharma) after seeding. After 24 h, 50 ng/ml TNFα (PeproTech) or 20 ng/ml RANKL (PeproTech) were added and cells differentiated for 5 to 6 days. On day 3, media were changed, leaving 120 µl of medium per well and adding 480 µl fresh medium including 187.5 µg/ml hPLEV-Fs, 50 ng/ml TNFα or 20 ng/ml RANKL. On days 5 and 6, cells were fixed using 10% FBS for 3 min and equal volumes of ethanol and acetone. After drying, cells were stained as described before [45] and washed with PBS (pH 7.4). Coverslips were mounted to microscopy slides with Mowiol (Mowiol 4–88, Carl Roth) and visual analysis was performed using light microscopy (Jenaval, Carl Zeiss) and a camera (AxioCam MRc5, Carl Zeiss). Experiments were performed in three independent biological replicates.

#### Osteoclastic resorption activity

A functional analysis of osteoclastic resorptive activity was performed using the Bone Resorption Assay Kit (Cosmo Bio Co. LTD) according to the manufacturer’s protocol. Briefly, cells were seeded in a concentration of 2.0 × 10^5^ cells per well in 500 µl phenol red free medium (Gibco MEM alpha (w/o Phenol red) (Gibco) + 10% FBS + 100 U/ml penicillin, 100 µg/ml streptomycin (Thermo-Fisher Scientific) + 1% M-CSF (Peprotech)) in a 48-well plate with a specific layer of calcium phosphate combined with a fluoresceinamine-labeled chondroitin sulphate (FACS) coating. Further, 12 h after seeding, hPLEV-F (Lysatpharma) treatment was started. After further 24 h, 50 ng/ml TNFα (PeproTech) or 20 ng/ml RANKL (PeproTech) were added. One media change was done 48 h after the TNFα or RANKL-stimulus. Activated osteoclasts dissolve the calcium phosphate and the FACS-layer is released into the supernatants. The fluorescence was measured on day 9 after seeding using a Tecan infinite 200Pro (Tecan Life Science) at excitation wavelength of 485 nm and emission wavelength of 527 nm. The experiment was performed in four independent biological replicates. Each biological replicate had two technical replicates. Fluorescence images of resorption pits on coated well-bottoms were taken using an inversed microscope (Primovert iLED Microscope, Carl Zeiss; Camera (EOS 77D, Canon)).

### Human osteoblasts

Collection of human oral bone was approved by the Institutional Review Board of the University Hospital Jena (No. 2019–1440-Material) and carried out according to The Code of Ethics of the World Medical Association (Declaration of Helsinki). All patients had previously signed informed consent.

#### Isolation and cultivation of human osteoblasts

**Fig. 7:**
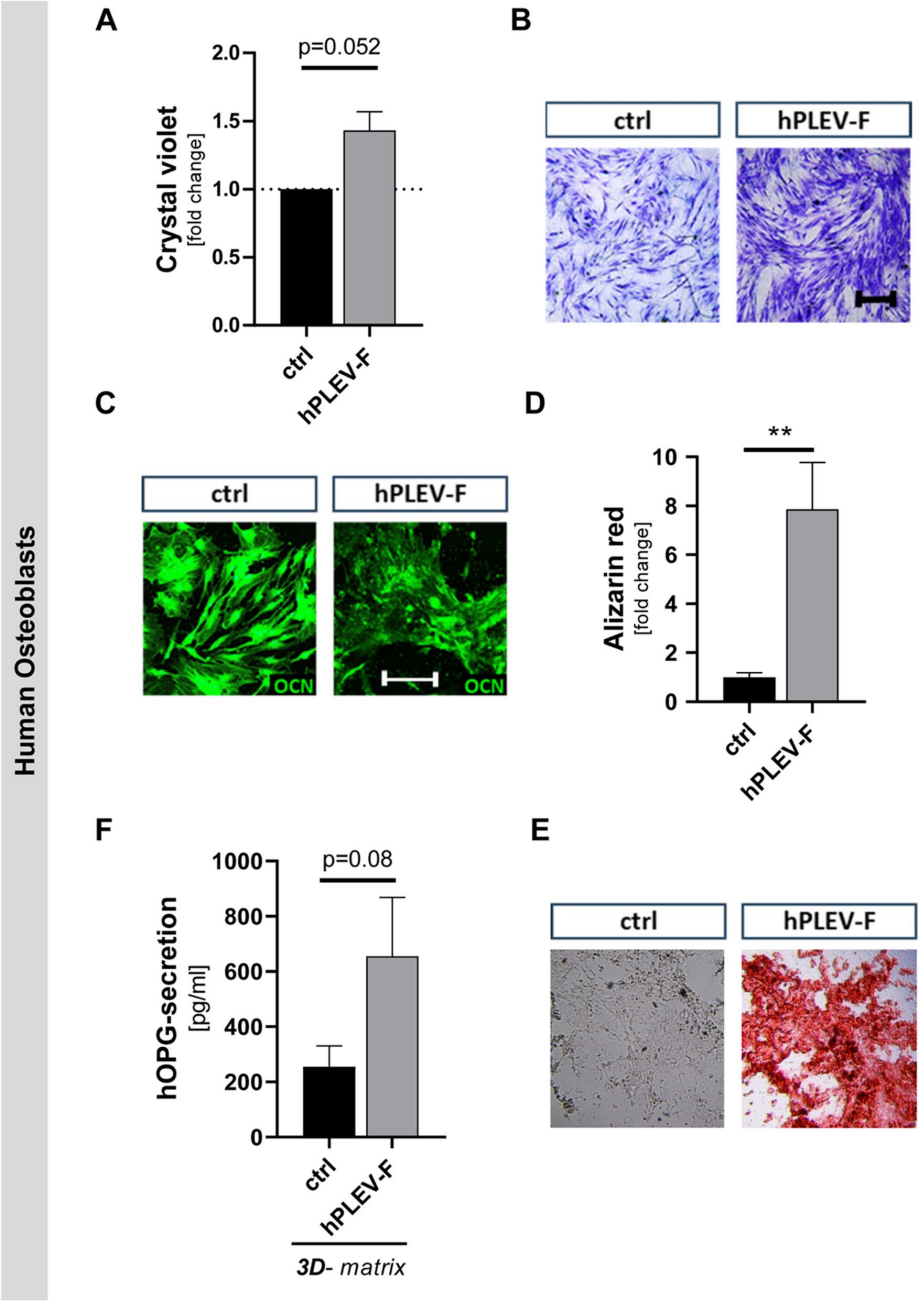
hPLEV-F-treatment enhances proliferation and differentiation as well as increases OPG-secretion from human osteoblasts seeded on β-TCPCM. **A**, **B** Primary human osteoblasts were cultured in hPLEV-F-supplemented proliferation medium and proliferation was quantified by **A** crystal violet staining. **B** After cell lysis, incorporated crystal violet was measured and depicted as fold change of control. **C**, **D** Osteoblasts were differentiated for 14 days under hPLEV-treatment and **C** stained and **D** quantified for mineralization in an alizarin red assay and calculated as fold change of control. **E** Additionally immunohistochemical staining for OCN was performed. **F** Secretion of hOPG as inhibitor of RANKL-mediated osteoclastogenesis was measured in an ELISA-assay after 14 days differentiation on resorbable ceramic-collagen foam matrix with incorporated β-tricalcium phosphate granules (β-TCPCM). *β*-*TCP* β-tricalcium phosphate, *OCN* osteocalcin, *hPLEV*-*F* human platelet lysate-derived extracellular vesicle fraction. Experiments were performed in at least three independent biological replicates. Statistical analyses: Student’s *t* test; ** p ≤ 0.01). Scale in **A** 200 µm and **C** 50 µm

For human osteoblast cultures, bone tissue was collected during dental implant procedures during the subsequent drilling steps. First, soft tissue was removed using a scalpel followed by two subsequent washing steps with PBS. If necessary, bone was cut in small pieces prior to mechanical deterioration by three 10 s vortex-steps. In between, bone fragments were allowed to settle down before PBS was exchanged. After the final washing step, tissue underwent collagenase digestion (375 U/ml Collagenase II in DMEM) for 2 h at 37 °C. Afterwards, bone fragments were washed three times in proliferation medium (DMEM low glucose (Thermo-Fisher Scientific)), 100 U/ml penicillin, 100 µg/ml streptomycin (Thermo-Fisher Scientific), 50 µg/ml gentamycin (Sigma-Aldrich), 1.25 µg/ml amphotericin B (Sigma-Aldrich) and transferred to a T25-flask with 5 ml proliferation medium. Medium was changed for the first time after 5 days of incubation. Subsequent media changes were performed three times a week until cells reached confluency. Cells were treated analogue to murine cells. For differentiation experiments, cells were seeded in a density 1.4 × 10^4^ cells per well on 24-well plates. After 3 days in culture, proliferation medium was exchanged for differentiation medium (proliferation medium supplemented with 100 µg/ml l-ascorbic acid, 10 mM β-glycerol phosphate and 100 nM dexamethasone) and hPLEV-Fs (Lysatpharma) were added. To analyse osteoblast differentiation, cells were incubated in differentiation media for up to 14 days. Cell culture medium was changed every 2 to 3 days. Supernatants were taken on day 14 before ending the experiment. Human osteoblast cultures were performed with at least three biological replicates derived from different donors. Each biological replicate had two technical replicates. Results from human osteoblast experiments are shown in Fig. 7.

### Gene expression analysis

RNA was isolated with TRIzol Reagent (Thermo Fisher Scientific)/1-bromo-3-chloropropane. Purification was performed using RNA Clean and Concentrator-5 kit (Zymo Research) according to the manufacturer’s instructions. The concentration and purity of RNA were measured with Nanodrop 2000 (Avantor). RNA was transcribed to cDNA with SuperScript IV Reverse Transcriptase (Thermo Fisher Scientific) according to the manufacturer’s protocol. For quantitative RTPCR gene expression levels, Luminaris HiGreen Master Mix (Thermo Fisher Scientific) was used and monitored by qTOWER3 (Analytic JENA). Gapdh (glyceraldehyde 3-phosphate dehydrogenase) and Rps29 (40S ribosomal protein S29) served as respective reference genes. Sequences of all primers are depicted in Additional file 1 Table 1.

Each primer pair was tested with template dilution series for the calculation of efficiency followed by the analysis of melting curve and agarose gel electrophoresis to exclude primer dimers. Analysis of data was performed using the ΔΔCT-method. The experiment was performed in three independent biological replicates, measured in duplicates.

### Enzyme-linked immunosorbent assay

Protein detection in murine and human samples was performed using Mouse osteoprotegerin (OPG)/TNFRSF11B Quantikine ELISA Kit (R&D Systems), Mouse Alkaline Phosphatase (ALP)/ab285274–ELISA Kit (Abcam) or Human TNFRSF11B (OPG)–ELISA Kit (Invitrogen) according to the manufacturer’s protocol. Secreted osteoprotegerin levels were calculated by comparison to a standard curve at an absorbance of 450 nm (Infinite M Nano plate reader, Tecan Life Science). Values were depicted as concentrations. The experiment was performed in three independent biological replicates and measured in duplicates.

### Multiplex immunoassay

hPLEV-F (Lysatpharma) and cell culture supernatants were analysed for protein contents in multiplex immunoassays. hPLEV-F were subjected to EV-lysis using an ultrasonic ice water bath at 35 kHz for 3 × 10 min. Protein detection was performed using LEGENDplex™ Human Bone Formation Panel (5-plex) w/VbP V02 (Biolegend) according to manufacturer’s protocol. OPG, PDGF-BB, alkaline phosphatase liver/bone/kidney (ALPL), Leptin and bone morphogenetic protein 2 (BMP-2) protein levels were determined using multiplex (Thermo-Fisher Scientific). Target levels were analysed using Novocyte Advanteo flow cytometry (Agilent). Additionally, regulated upon activation, normal T cell expressed and secreted (also known as CCL5) (RANTES) protein detection was performed using ELISA MAX™ Deluxe Set Human CCL5 (RANTES) (Biolegend) (Fig. 2D). Secreted RANTES levels were measured using the endpoint method, spiral average (12 points) at an absorbance of 450 nm (Spectrostar Nano, BMG). Protein content in lysed hPLEV-F was determined in relation to an EV-Fraction (EV-F) of pooled human plasma (commercially purchased by Institute of clinical Transfusion medicine Jena GmbH). The experiment was performed in at least three independent biological replicates.

Protein detection in cell culture supernatants was performed using Mouse ProcartaPlex Mix & Match 8-Plex (Invitrogen) according to the manufacturer’s protocol (Figs. 2H, J–O, 3G–N and 5). In the multiplex immunoassay, we targeted interleukins (IL) and chemokines: IL-1β, IL-10, IL-33, IL-6, M-CSF, MIP2α ((macrophage inflammatory protein 2-alpha) = CXCL2 (Chemokine (C-X-C motif) Ligand 2)), RANKL and TNFα. Secreted target levels were analysed using Bio-Plex (Bio-Rad). Concentrations were determined by comparing values to a standard curve. The experiment was performed in three independent biological replicates.

### Statistical analyses

Pairwise comparisons were performed with two-tailed Student’s *t* test. Differences between more than two groups were assessed using two-way ANOVA and post hoc test (Tukey). All values are presented as mean ± SE. Differences between groups were considered statistically significant at *p* < 0.05. Analyses were performed in GraphPad/InStat3 (GraphPad Software Inc.). For exclusion of single data points, Grubb’s outlier test was performed.

## Results

### hPLEV-Fs regulate phosphorylation activity on bone-specific targets

To determine influence of hPLEV-F on osteoblasts under basal and pro-inflammatory conditions, primary murine osteoblasts were investigated for changes in their phosphoproteome in association with hPLEV-F and in the presence of pro-inflammatory TNFα (*ctrl/ctrl*, *ctrl/hPLEV*-*F*, *TNFα/ctrl* and *TNFα*/*hPLEV*-*F*). Principal component analysis (PCA) revealed that all conditions induced similar protein compositions (Fig. 1A) and phospho-enrichment profiles (Fig. 1B), with no noticeable changes attributable to hPLEV-F or TNFα treatment. However, differential protein expression analysis, depicted as Volcano Plots (Fig. 1C, D), revealed increased expression (depicted in red) of *Hmox1* (*Heme oxygenase 1*), *Ptgs2* (*Prostaglandin*-*endoperoxide synthase 2*), *Itga2* (*Integrin alpha 2*) and *Ankh* (*Progressive ankylosis protein homolog*), while *Col12a1* (*Collagen type XII alpha 1 chain*), *Cemp1* (*Cementum protein 1*), *S100a6* (*S100 calcium*-*binding protein A6*) and *Gas6* (*Growth arrest*-*specific 6*) (in blue) showed reduced expression. Upon additional TNFα treatment, *Ptgs2* and *Ankh* remained consistently upregulated, with increased expression of *Parvb* (*Parvin beta*) and *Inhba* (*Inhibin beta*). A *Col12a1* expression was unaffected by TNFα exposure and reduced, along with *Mfge8* (*Milk fat globule*-*EGF factor 8 protein*) and *Col1a2* (*Collagen type I alpha 2 chain*) (Fig. 1D).

Downstream analysis identified a group of upregulated and downregulated kinases based on the top three phosphosites, predicted to influence kinase activity through their flanking regions. Among these, several key players of bone metabolism, such as mitogen-activated protein kinase 14/p38 alpha (MAPK14/p38α) (Fig. 1E, F, H), mitogen-activated protein kinase 3/extracellular signal-regulated kinase 1 (MAPK3/ERK1) (Fig. 1E, G, H), mitogen-activated protein kinase 1/extracellular signal-regulated kinase 2 (MAPK1/ERK2) (Fig. 1E, F, G), AKT1 (protein kinase B) (Fig. 1E, H), and cyclin-dependent kinase 5 (CDK5) (Fig. 1E, F, G, H) were identified. In the over-representation analysis (ORA) of the enriched data set, several pathways were significantly enriched in both comparisons (ctrl/ctrl vs. ctrl/EV and TNFα/ctrl vs. TNFα/EV), demonstrating an influence of hPLEV-F under basal and pro-inflammatory conditions. Further, the enrichment ratio demonstrated association with specific Gene Ontology terms or particular pathways (KEGG Pathway/Reactome Pathway) (Fig. 1I). Overall, phosphoproteome analyses indicated regulation of proteins involved in osteoblast proliferation, differentiation, and inflammatory response, in addition to key kinases related to these processes.

### hPLEV-F enhance osteoblast proliferation and differentiation

Based on the phosphoproteome results predicting influence of hPLEV-Fs on osteoblast metabolism, and response to an inflammatory trigger, the direct impact of hPLEV-F on osteoblastic proliferation, differentiation and immunological functions in inflammatory conditions was investigated.

Primary murine osteoblasts were pretreated with hPLEV-F and incubated with TNFα to mimic a pro-inflammatory microenvirononment. Osteoblast proliferation was determined in the respective treatment groups (± TNFα, ± hPLEV-F) and compared to controls using crystal violet staining (Fig. 2A, B). hPLEV-F increased osteoblastic crystal violet staining compared to controls, indicating a greater cell number in response to hPLEV-F (*p = 0.02). TNFα-stimulated cultures with or without hPLEV-F show similar tendencies indicating proliferation is unaffected by TNFα-treatment (ctrl/ctrl vs. TNFα/hPLEV-F: *p* = 0.07; ctrl/hPLEV-F vs. TNFα/ctrl: *p* = 0.05) (Fig. 2A, B). To investigate whether hPLEV-F contain relevant bone metabolism factors that could support the depicted osteoblastic phenotype, the protein content of various effectors in hPLEV-F was quantified. As shown in Fig. 2, hPLEV-F contain enhanced platelet-derived growth factor BB (PDGF-BB) (Fig. 2C) and regulated upon activation, normal T cell expressed and secreted (also known as CCL5) (RANTES) levels (Fig. 2D). Further, hPLEV-F content of bone morphogenetic protein 2 (BMP-2) and Leptin was at approximately 50 pg/mg protein comparable to the respective control plasma fractions (Additional file 2: Fig. S1A, B).

Next, osteoblasts were differentiated in the absence and presence of hPLEV-Fs to determine their influence on osteoblastic differentiation. hPLEV-F itself contain very low (approximately 18 pg/ml) levels of alkaline phosphatase (liver/bone/kidney—ALPL) (Fig. 2E). However, ALP content significantly increased in the cell culture supernatant of hPLEV-F-treated osteoblasts (*p = 0.02) (Fig. 2F) compared to the originally added ALPL within the hPLEV-F (Fig. 2F, dashed line), showing about a 1.7-fold increase compared to controls at day seven of osteoblast differentiation (Fig. 2F). Moreover, at 14 days in differentiation culture, photometric quantification of alizarin red staining revealed a threefold increase of alizarin red staining in hPLEV-F-treated cells compared to controls (**p = 0.001) (Fig. 2G), which was stably reproducible when using different hPLEV-batches (Additional file 2: Fig. S2). However, when TNFα was concomitantly applied to osteoblast cultures, hPLEV-F-induced effects on mineralization were abolished (Fig. 2G).

Further, hPLEV-Fs enhanced OPG-secretion into cellular supernatant (Fig. 2H), leading to much higher yields than those applied with hPLEV-Fs (Fig. 2I). Additional multiplex immunoassay analysis revealed that enhanced mineralization activity was accompanied by changes in the cellular secretory pattern (Fig. 2J–O). TNFα-, IL-6-, IL-10- and MIP2α-secretion (Fig. 2J–M) were significantly increased under hPLEV-F-treatment, while other markers—M-CSF, IL-33 (Fig. 2N, O) and IL-1β (data not shown)—did not change significantly. Further, no soluble RANKL was detectable in the supernatants of all conditions (data not shown).

### hPLEV-F balances inflammation and bone remodeling factors in osteoblasts

To investigate the direct influence of hPLEV-F on osteoblastic differentiation markers during the differentiation process, their expression was analysed in response to hPLEV-F treatment after six initial days without hPLEV-F. Expression levels appeared either unaffected (collagen type I alpha 1 (*Col1A1*)) or even reduced (*Runx2* (*p = 0.025), Osteocalcin (*Ocn*) (***p = 0.0002)) by hPLEV-F-treatment (Fig. 3A–C). Further, expression of osteoclast differentiation-inhibitor *Opg* was significantly decreased (****p < 0.0001) (Fig. 3D). Though *Rankl* showed no significant change in expression (Fig. 3E), *Tnf*-levels were significantly increased following hPLEV-F-treatment (**p = 0.002) (Fig. 3F). In contrast, application of TNFα reduced expression levels of differentiation markers (*Col1A1*, *Runx2*, *Ocn*, *Opg*; Fig. S3A–D), which remained unaffected by hPLEV-F-application. Moreover, TNFα-modulated expression of factors active in osteoclastic differentiation (*Rankl*, *Tnf*; Fig. S3E,F) also remained unchanged upon hPLEV-F-stimulation. In contrast, associated cell culture supernatants exhibited no basal TNFα-secretion. However, when TNFα was added to mimic a pro-inflammatory condition, TNFα-levels significantly increased compared to basal level (ctrl/ctrl vs. TNFα/ctrl: *p = 0.0158). Of note, when hPLEV-F was applied, TNFα-levels were reduced to concentrations not significantly different from controls (Fig. 3G). Furthermore, TNFα-treatment significantly enhanced IL-1β-secretion, whereas concomitant addition of hPLEV-F reduced it (ctrl/TNFα vs. hPLEV-F/TNFα: *p = 0.0216) (Fig. 3H). hPLEV-F-prestimulation appeared to reduce IL-33 (Fig. 3I) and IL-10 (Fig. 3J), though, there were only significant differences between ctrl/hPLEV-F and TNFα/ctrl. Pertaining to IL-6-secretion, the increase in ctrl/hPLEV-F and TNFα/hPLEV-F cultures as compared to hPLEV-F-untreated controls was not significant (Fig. 3K). Additionally, hPLEV-F-treatment did not regulate MIP2α-secretion (Fig. 3L). Osteoclastogenic M-CSF secretion increased under TNFα-treatment and was unaffected by addition of hPLEV-F (Fig. 3M); however, hPLEV reduced osteoblastic RANKL-secretion under inflammatory conditions (*p = 0.0190) (Fig. 3N).

### hPLEV-F-treatment of osteoblasts improves attachment, differentiation and secretion of osteoclast-inhibiting factors in an alloplastic bone graft matrix

hPLEV-Fs increased osteoblastic bone mineralisation activity in depicted in vitro experiments. In consideration of future in vivo applications, a three-dimensional resorbable ceramic-collagen foam matrix containing β-tricalcium phosphate granules (β-TCP in ceramic-collagen matrix—β-TCPCM) (CERASORB® Mouldable Foam, Curasan AG) was used to investigate impact of hPLEV-F as a biologizing agent. Primary murine osteoblasts were seeded either directly (2D plate) or on the β-TCP-matrix (3D-matrix) (Fig. 4A). hPLEV-F-treatment doubled the number of cells settled on the surface of the collagen matrix as depicted in DAPI-stained nuclei (Fig. 4B, upper lane, depicted in black on white background, Fig. 4C). Further, hPLEV-F increased osteocalcin-staining (OCN—green in Fig. 4B, lower lane, DAPI depicted in blue) compared to controls (****p < 0.0001) (Fig. 4D). This was accompanied by a significant increase in OPG-secretion (***p = 0.0007) (Fig. 4E). Of note, hPLEV-F-induced increase of secretory OPG was more pronounced in 3D-matrix cultures upon hPLEV-F-treatment (‘3D’) as compared to cells seeded without foam on a cell culture bottom (‘2D’) (*p = 0.0201) (Fig. 4F; both depicted as fold change of respective ctrl).

### Osteoblasts seeded on alloplastic bone graft matrix demonstrated a reduced inflammatory phenotype under hPLEV-F-treatment

Based on the synergistic effects of β-TCPCM and hPLEV-F-treatment observed during osteoblastic differentiation, the impact of combined β-TCPCM/hPLEV-F-application on the secretome was subsequently characterized. Bone homeostasis is maintained through a delicate balance between bone formation and degradation that is regulated by the reciprocal influence of osteoblasts and osteoclasts. This process involves the differential release of various inflammatory and bone-homeostatic cytokines [51]. At early differentiation time points, cytokine secretions appeared rather low with only few differences in 2D (cell culture bottom)- and 3D (β-TCPCM)-cultures (Fig. 5A–G, left). In this context, a reduced inflammatory response mediated by MIP-2α (Fig. 5A) was detectable in cells on β-TCPCM, particularly under the influence of hPLEV-F (Fig. 5A), whereas IL-6-levels appeared lowest under 2D control conditions (Fig. 5B).

At later differentiation stages, differences between 2D- and 3D-cultures became more pronounced with reduced secretion of all measured inflammation-regulating cytokines in hPLEV-F-treated groups on β-TCPCM (Fig. 5A–G, right). In this context, there were consistent changes in terms of the secretion of pro-inflammatory, bone-resorptive cytokines (IL-1β, TNFα) (Fig. 5C, D) and anti-inflammatory, bone-protective mediators (IL-33, IL-10) (Fig. 5E, F). Additionally, secretion of macrophage-stimulating M-CSF was increased (Fig. 5G), whereas soluble RANKL appeared below detection limits in all groups (data not shown).

### hPLEV-Fs inhibit RANKL- and TNFα-mediated osteoclast differentiation and resorption activity

hPLEV-F increased osteoblastic mineralization activity accompanied by an increase in secreted RANKL-intercepting OPG and a decrease in TNFα-secretion. To investigate a direct impact of hPLEV-Fs on osteoclast differentiation and functionality, primary murine bone marrow cells (BMC) as osteoclast precursors were treated with hPLEV-F. hPLEV-F increased *Trap*-expression (**p = 0.0024), whereas expression levels of *Rank* and *Tnf* remained unaffected compared to controls (Fig. 6A). However, continuous application of hPLEV-F in parallel to osteoclastic differentiation factors TNFα- or RANKL abolished TRAP^+^ cells in number and size, leading to fewer TRAP^+^ mononuclear cells in hPLEV-F compared to controls (Fig. 6B). To analyse osteoclastic resorption activity, BMC were seeded on culture plates coated with fluorescent calcium phosphate that can be detected in the supernatant as a measure of resorption activity. After osteoclastic differentiation, resorption pit formation was analysed using light microscopy (Fig. 6C) and fluorescence intensity determined in the supernatant (Fig. 6D). Osteoclastogenic stimulation of BMCs induced formation of resorption pits (Fig. 6C, arrows) that were not detectable in non-stimulated control-conditions and upon addition of hPLEV-F. In line, application of hPLEV-Fs to M-CSF/RANKL- or M-CSF/TNFα-stimulated cultures significantly reduced liberated fluorescent calcium phosphate (RANKL ctrl vs. hPLEV-F: *p = 0.0358; TNFα ctrl vs hPLEV-F: * *p* = 0.0329), comparison to respective hPLEV-F negative control) (Fig. 6D).

### hPLEV-Fs increases proliferation and differentiation of human osteoblasts

Similar to murine osteoblasts, hPLEV-F increased cell density as visible in crystal violet staining in primary human osteoblast cultures (Fig. 7A). Quantification revealed a nearly 1.5-fold increase in cell number after application of hPLEV-F (Fig. 7B). Although osteocalcin staining appeared similar in both conditions (Fig. 7C), we detected an increase in mineralization using a quantitative alizarin red assay in osteoblast cultures differentiated for two-weeks in the presence of hPLEV-F (**p = 0.0051) (Fig. 7D, E). To investigate the regulatory function of osteoblasts in the presence of hPLEV-F, secretion of RANKL-binding OPG was analysed in the supernatants. Fourteen days post differentiating stimulus, total amount of secreted hOPG was significantly increased in hPLEV-F-treated 3D-cultures compared to controls (Fig. 7F).

## Discussion

Previously, it had been indicated that human platelet lysate [52] and EV-preparations - particularly those originating from mesenchymal cells - influence bone healing [53]. Therefore, we analysed hPLEV-F in the context of cellular key players in bone metabolism, specifically focusing on functionality of bone-forming and bone-degrading cells and their interactions. Our findings demonstrate the potential of hPLEV-F to enhance bone regeneration as they promote bone cell proliferation and mineralization when applied to osteoblasts. Additionally, hPLEV-F reduces formation of bone-degrading osteoclasts. The use of a β-TCP containing ceramic-collagen matrix (β-TCPCM) further enhanced the impact of hPLEV-F on cellular attachment and osteocalcin marker expression accompanied by increased cellular OPG-secretion, suggesting that this combination could improve the rehabilitation of bone defects.

In response to hPLEV-F treatment, phosphoproteomic screening revealed only isolated osteoblastic changes and associated principal component analysis (PCA) did not show significant differences. Of note, additional application of TNFα did not induce notable changes at the time of analysis. In contrast, human periodontal ligament stem cells (hPDLSCs) demonstrated marked changes in phosphoproteomic composition after a 30-min TNFα-incubation [54]. This indicates a certain cell-specificity in inflammatory conditions. Nevertheless, ORA analysis revealed that hPLEV-F treatment was associated with changes in wound healing and bone remodeling pathways. Further, identification of specific phosphorylation sites enabled the prediction of differential kinase expression. The identified kinases included several candidates previously linked to signalling cascades in bone metabolism. In addition to MAPK14, which is known to play a role in various cell differentiation processes and specifically stimulates osteoblast differentiation [55], we observed indications of increased CDK5 levels. CDK5 also promotes osteoblast differentiation and aids in the healing of bone fractures [56]. In this context, kinases ERK1 and ERK2 emerged as well, both of which are essential for osteoblast differentiation [57]. Previously published data showed the application of platelet-derived apoptotic vesicles to increase osteogenesis in mesenchymal stem cells via AKT-signalling [58]. In line, our phosphoproteomic analyses revealed increased expression of AKT1, a kinase known to influence both osteoblast and osteoclast activity in bone metabolism [42] identifying this kinase as a potential target. However, the precise pathway in this context remains to be elucidated. Consistently, in vitro experiments demonstrated that the application of hPLEV-F enhanced proliferation and mineralization in both murine and human osteoblasts. This result aligns with previously published data showing similar effects of platelet preparations on human mesenchymal stem cells (MSC) [41].

Previous investigations have shown that bone-targeted platelet-derived exosomal preparations (PL-exo-Aln) improve dexamethasone-inhibited osteogenesis in BMCs in vitro and alleviate glucocorticoid-induced osteoporosis in vivo [35]. In line, our results demonstrated that hPLEV-F treatment of murine and human primary osteoblasts not only mitigates the inhibitory effects reported by Zheng et al. [35], but also enhances osteoblast mineralization. Although, inflammatory TNFα appeared to diminish this enhancement.

Screening of lysed hPLEV-F for potential bone-influencing mediators revealed elevated levels of RANTES and PDGF-BB. Previous studies have shown that RANTES can enhance osteogenic differentiation of human mesenchymal stem cells in a dexamethasone-dependent manner [59]. Since RANTES is an inflammatory cytokine with a range of functions mediated through different receptors, its specific role in the current context requires further investigation. PDGF-BB has previously been described to promote proliferation of human osteoblasts [60] and improve osteogenic differentiation in various cell types [61], e.g., in bone marrow stromal cells (BMSC) [62] or human osteoblasts [63]. However, PDGF-BB has been shown to negate the osteogenic effects of BMP-2 in mesenchymal progenitor cells through a PDGFRβ-dependent mechanism [64]. In contrast to previously published data, our analysis of lysed hPLEV-F revealed that BMP-2 was present in only negligible amounts, suggesting that PDGF-BB could be a factor in the detected osteoblast differentiation. PDGF-BB acts via the JAK2/Src pathway as demonstrated in MC3T3-E1 cells [63] and in an ERK-dependent manner as shown in cells derived from diabetic rats, where it promoted proliferation and osteogenic differentiation [65]. This finding aligns with our phosphoproteome-depicted kinase regulation, which showed that ERK1 and ERK2 were upregulated in hPLEV-F-treated osteoblasts. Although hPLEV-F treatment appeared to enhance mineralization in murine and human osteoblasts, gene expression analysis of pre-differentiated osteoblasts suggested that effects of hPLEV-F might be restricted to certain stages of cellular development. Specifically, hPLEV-F treatment at more advanced developmental stages resulted in reduction of osteoblast differentiation genes. Interestingly, we observed that pro-inflammatory and osteoclastogenic mediators were regulated alongside anti-inflammatory and osteogenic markers, suggesting a mechanism that maintains a balance between inflammatory processes and bone remodelling. With regards to TNFα in particular, different effects have been shown depending on cellular differentiation and inflammation status. Although high levels of TNFα inhibit osteoblast differentiation, low levels promote osteoblast differentiation of human dental pulp stem cells [66]. In endothelial cells, activated monocytes-derived extracellular vesicles carrying mitochondria induced a TNFα- response [67, 68]. Of note, platelet-derived extracellular vesicles could also carry mitochondria [69]. In line, we detected enhanced TNFα-secretion upon hPLEV-F-stimulation. Whether this is similar to mechanisms described in endothelial cells remains to be elucidated [67, 68].

Osteoblastic cytokine secretion changes in the course of cellular differentiation [58]. Accordingly, hPLEV-F treatment might exercise a differential impact in relation to the developmental stage and inflammatory environment. Previous studies demonstrated platelet-derived EV-preparations act pro- or anti-inflammatory depending on the cellular context [70]. In line, the application of hPLEV-F to osteoblasts results in a seemingly contradictory picture: during osteoblastic differentiation, not only osteoconductive cytokines (IL-10, MIP2α) are upregulated, but also signalling molecules better known for their inflammatory roles due to their involvement in osteoclast differentiation (TNFα, IL-6). We therefore suspect that hPLEV-F treatment has a rather dual effect, which promotes regeneration while simultaneously preventing an excessive response through concurrent counter-regulatory mechanisms. Overall, bone regeneration is regulated by balanced interaction of inflammatory cytokines and a non-resolving hyperinflammatory response - as for instance observed in periodontitis - could lead to destruction of bone tissue [71]. Moreover, hPLEV-F exhibits an inflammation-reducing impact under an inflammatory TNFα stimulus. However, as observed in differentiation-dependent secretion patterns, this appears with both pro- and anti-inflammatory cytokines in the same manner, most likely maintaining inflammatory balance.

hPLEV-F treatment reduced TNFα-secretion back to baseline following TNFα application. While the precise mechanism remains unclear, this reduction in TNFα was accompanied by decreases in all investigated cytokines except IL-6 and MIP2α, suggesting a potential role for hPLEV-F in maintaining a physiological balance. In this context, we observed a reduction in osteoclastogenic factors such as TNFα, M-CSF, and RANKL, indicating that hPLEV-F treatment might reduce inflammation-triggered bone loss. Although hPLEV-F treatment induced a reduction in RANKL secretion during the course of differentiation, its gene expression levels remained unaltered. RANKL undergoes post-translational cleavage by specific proteases to generate a soluble isoform capable of functioning as an effector molecule. Nonetheless, the membrane-bound form of RANKL also promotes osteoclastogenesis and is therefore of considerable significance in co-culture systems and in vivo models [72].

Additionally, the increased release of OPG associated with hPLEV-F treatment suggests enhanced osteoblast-osteoclast interactions. Previous studies demonstrated that treatment with platelet-derived exosomes can elevate osteoblast OPG-levels and improve coupling between bone formation and angiogenesis [35]. Although our data support the maintenance of an inflammatory equilibrium, we observed a shift towards bone formation. Even though an acute hPLEV-F-stimulation induced a reduction in expression of osteoblastic differentiation factors, a prolonged treatment enhanced osteoblast mineralization but also reduced the secretion of TNFα and RANKL. Additionally, it led to increased release of RANKL-binding OPG.

In line with its osteoconductive properties [11], the simultaneous use of the β-TCPCM with the hPLEV-F further increased osteoblast differentiation. Additionally, as the cells progressively mature - particularly when used in combination with the matrix - their inflammatory potential decreases, potentially reducing the overall rate of inflammation-driven bone metabolism and in the course of this, to support regeneration process.

Besides the impact of hPLEV-F-treatment on osteoclasts, hPLEV-F directly affected osteoclast differentiation. Similar to data provided by Zheng et al. [35] describing modified PL-derived exosomes as inhibitors of osteoclast differentiation, we showed that hPLEV-F-treatment reduced osteoclast differentiation. Our data indicate that treatment with hPLEV-F inhibits initial differentiation steps of bone marrow cells undergoing transformation to *Trap*-expressing osteoclast-precursors. Thereby, addition of TNFα and RANKL failed to induce further cellular development of hPLEV-F-challenged cells; leaving them at a stage of mononuclear TRAP^+^ cells without resorptive activity. Our data from osteoblast cultures suggest the potential of hPLEV-F for direct intervention in cellular mechanisms, which might also apply to osteoclastic pathways and could be elucidated in future studies. In addition to the involvement of various proteins and signalling molecules stored in extracellular vesicles, different microRNAs (miRNAs) may also play a role in the observed processes [32] and could be evaluated.

Limitation of the current study might pertain to the single-cell cultures. However, we purposely investigated impact of hPLEV-F in a cell-targeted manner to outline the potential of hPLEV-F and investigate combination of hPLEV-F and β-TCPCM. Based on our results depicting a synergistic effect of hPLEV-F and β-TCPCM, a targeted coupling of the hPLEV-F to the bone substitute material might be a promising solution to biologize current alloplastic materials. Consistent with findings of Yuste et al. [73], osteoblasts in the 3D scaffold exhibited improved attachment and enhanced proliferation. In a direct comparison between 2 and 3D scaffolds, both treated with hPLEV-F, OPG secretion was significantly higher in the 3D culture. This is consistent with previous findings demonstrating a higher osteogenic capacity in 3D compared to 2D cultures [74]. Wang et al. reported promising results with their Ost-EVs@mHA/PEG hydrogel system [75], although previous studies highlighted that conventional hydrogels often have suboptimal mechanical properties, stability and cell adhesion [53]. In contrast, the ceramic-collagen matrix has been shown to provide a suitable microenvironment for osteoblast and osteocyte cultivation and to facilitate the differentiation process [76]. Moreover, the β-TCP granules incorporated into the matrix may further enhance osteoblast proliferation, differentiation and mineralization, as local Ca^2+^ concentration has been shown to positively impact osteoblasts in 3D cultures [77]. We utilized a novel approach by applying hPLEV-Fs, known for their bone regeneration-promoting properties, in a β-TCPCM, a material already being used for bony defects in dental and orthopaedic fields. The implantation of foreign material typically elicits an immune response [78]. In the context of bone metabolism, this could also be advantageous, as bone regeneration is modulated by inflammatory processes. However, excessive inflammation (hyperinflammation) may result in increased bone resorption [71]. Previous studies have shown that not only the use of platelet-derived EVs [79] but also the application of beta-TCP [80] influences inflammatory processes by promoting M2 macrophage polarization. Our in vitro data suggest that it promotes a balance between pro- and anti-inflammatory signalling and supports osteoblastic differentiation. However, in the present context, we have only quantified a limited selection of cytokines. In future studies - especially in vitro investigations - this selection should be expanded. Nevertheless, the data also indicate that a single application may have adverse effects on osteoblastic differentiation. This could necessitate repeated administration or, alternatively, a formulation with sustained-release (depot) properties. In addition, the here used hPLEV-F-preparation inhibits osteoclast differentiation, thereby potentially decreasing their promotional effects on osteoblast differentiation [80]. While the vesicles may suppress necessary immune responses and delay healing, our results suggest that they help prevent hyperinflammation by restoring balance, though these complex immune interactions might require further in vivo investigation.

Conducting the study in murine cells was essential, as murine models are also used in in vivo studies. However, since the transferability to a human system had to be ensured, the most important experiments directly aimed at bone formation were repeated in human primary cells. Moreover, to address the complex physiological microenvironment of the human body, future studies could consider co-culturing osteoblasts with bone marrow cells, osteoclasts, osteocytes, fibroblasts and other bone-lining cells to more accurately mimic in vivo conditions and provide a holistic understanding of hPLEV-F effects on bone regeneration allowing translation and increasing benefits for a clinical setting.

## Conclusions

Our results demonstrate that hPLEV-F are candidates for enhancing bone regeneration capacity of a β-TCP-collagen matrix at the cellular interface by improving new bone formation and reducing bone resorption; thereby being a potential candidate for future clinical applications. The three-dimensional structure in combination with hPLEV-F creates a bioactive scaffold with a more than threefold improved osteogenic capacity, highlighting the significant potential of this combination. This opens up innovative possibilities in bone regeneration by suggesting novel biointerface characteristics - thereby addressing challenges associated with complex regenerative applications and advancing patient care in the future.

## Supplementary Information

Additional file 1: Table 1–Table 1. Murine qRT-PCR-Primer.

Additional file 2: Figures S1, S2, S3–Fig. S1- Lysates of human platelet lysate derived extracellular vesicle fractionsdo not contain enhanced levels of Leptin and BMP2. Fig. S2–Different hPLEV-F-preparations exert comparable effects on osteoblast mineralization activity. Fig. S3–Under inflammatory conditions, hPLEV-F have no impact on expression levels of bone regulatory proteins in osteoblast.

## Data Availability

The authors declare that the main data supporting the finding of this study are included in this published article and raw data from this study are accessible upon request.

## Abbreviations

ACN: Acetonitrile
AKT1: Protein kinase B
ALP: Alkaline phosphatase
ALPL: Alkaline phosphatase liver/bone/kidney
Ankh: Progressive ankylosis protein homolog
ANOVA: Analysis of variance
Bglap2: Bone gamma-carboxyglutamate protein 2 (also known as Osteocalcin)
BMC: Bone marrow cells
BMP: Bone morphogenetic protein
BMP-2: Bone morphogenetic protein 2
BMSC: Bone marrow stromal cells
C57B16: A strain of laboratory mouse (commonly referred to as C57BL/6)
CCL5: Chemokine (C–C motif) ligand 5 (same as RANTES)
CD63: Cluster of differentiation 63 (another tetraspanin marker on extracellular vesicles)
CD9: Cluster of differentiation 9 (a tetraspanin protein commonly found on extracellular vesicles)
CDK5: Cyclin-dependent kinase 5
cDNA: Complementary DNA
Cemp1: Cementum protein 1
Col12a1: Collagen type XII alpha 1 chain
Col1A1: Collagen type I alpha 1 chain
Col1a2: Collagen type I alpha 2 chain
ctrl: Control
CXCL2: C-X-C motif chemokine ligand 2
DAPI: 4',6-Diamidino-2-phenylindole, dihydrochloride (core staining)
ddH_2_O: Double-distilled water
DIA: Data-independent acquisition
DMEM: Dulbecco's Modified Eagle's Medium
DTT: Dithiothreitol (a reducing agent)
EDTA: Ethylenediaminetetraacetic acid
ELISA: Enzyme-linked immunosorbent assay
ERK: Extracellular signal-regulated kinase
EV: Extracellular vesicle
EV-F: Extracellular vesicle fraction
FA: Formic acid
FACS: Fluoresceinamine-labeled chondroitin sulphate
FBS: Fetal bovine serum
FDR: False discovery rate
Fe(III)-IMAC: Iron (III) immobilized metal affinity chromatography
FWHM: Full width at half maximum
Gapdh: Glyceraldehyde 3-phosphate dehydrogenase
Gas6: Growth arrest-specific 6
Gene ID: Gene identifier
HEPES: 4-(2-Hydroxyethyl)-1-piperazineethanesulfonic acid (a buffering agent)
Hmox1: Heme oxygenase 1
hOB: Human osteoblast
hOPG: Human osteoprotegerin
hPDLSC: Human periodontal ligament stem cells
hPLEV: Human platelet lysate-derived extracellular vesicles
hPLEV-F: Human platelet lysate-derived extracellular vesicle fraction, fraction enriched for human platelet lysate-derived extracellular vesicles
HSP70: Heat shock protein 70
HSS: High strength silica
IgG: Immunoglobulin G
IL-10: Interleukin-10
IL-1β: Interleukin-1 beta
IL-33: Interleukin-33
IL-6: Interleukin-6
Inhba: Inhibin beta A
iRT: Indexed retention time
Itga2: Integrin alpha 2
KEGG: Kyoto Encyclopedia of Genes and Genomes
LC: Liquid chromatography
LC-MS: Liquid chromatography–mass spectrometry
m/z: Mass-to-charge ratio
MAPK1/ERK2: Mitogen-activated protein kinase 1/extracellular signal-regulated kinase 2
MAPK14/p38α: Mitogen-activated protein kinase 14/p38 alpha
MAPK3/ERK1: Mitogen-activated protein kinase 3/extracellular signal-regulated kinase 1
MC3T3-E1: Mouse osteoblast precursor cell line
M-CSF: Macrophage colony-stimulating factor
MEM: Minimum essential medium
Mfge8: Milk fat globule-EGF factor 8 protein
MIP2α: Macrophage inflammatory protein 2 alpha
miRNAs: MicroRNAs
mOB: Murine osteoblast
MS: Mass spectrometry
MSC: Mesenchymal stem cells, bone marrow stromal cells
NaCl: Sodium chloride
NCBI: National Center for Biotechnology Informtion
NGS: Normal goat serum
NTA: Nanoparticle tracking analysis
OCN: Osteocalcin
OPG: Osteoprotegerin
ORA: Over-representation analysis
Ost-EVs@mHA: Osteoblast extracellular vesicles at modified hydroxyapatite
Parvb: Parvin beta
PBS: Phosphate-buffered saline
PCA: Principal component analysis
PDGF-BB: Platelet-derived growth factor BB
PDGFRβ: Platelet-derived growth factor receptor beta
PEG: Polyethylene glycol
PFA: Paraformaldehyde
pH: Potential of hydrogen
PHB: Prohibitin
PL: Platelet lysate
PRF: Platelet-rich fibrin
PRP: Platelet-rich plasma
Ptgs2: Prostaglandin-endoperoxide synthase 2
PTM: Post-translational modification
RANK: Tumor necrosis factor receptor superfamily, member 11a (activator of NF-kB)
RANKL: Tumor necrosis factor (ligand) superfamily, member 11 (receptor activator of NF-kB-ligand)
RANTES: Regulated upon activation, normal T cell expressed and secreted (also known as CCL5)
RNA: Ribonucleic acid
Rps29: 40S ribosomal protein S29
Runx2: Runt-related transcription factor 2
SDS: Sodium dodecyl sulfate
SYN: Syntenin
S100a6: S100 calcium-binding protein A6
TEAB: Triethylammonium bicarbonate (buffer in mass spectrometry)
TFA: Trifluoroacetic acid
TGF-β1: Transforming growth factor beta 1
Tnfrsf11a: Tumor necrosis factor receptor superfamily, member 11a (activator of NF-kB)
TNFRSF11B: Tumor necrosis factor receptor superfamily, member 11B (another name for osteoprotegerin)
Tnfrsf11b: Tumor necrosis factor receptor superfamily, member 11b
Tnfsf11: Tumor necrosis factor (ligand) superfamily, member 11 (receptor activator of NF-kB-ligand)
TNFα: Tumor necrosis factor alpha
TRAP: Tartrate-resistant acid phosphatase
TSG101: Tumor susceptibility gene 101 protein (involved in vesicle formation)
TX-100: Triton X-100
VEGF: Vascular endothelial growth factor
Wnt: The wingless-related integration site pathway
x g: Times gravity (g-force) for centrifugation
αMEM: Alpha minimum essential medium
β-TCP: β-tricalcium phosphate
β-TCPCM: β-tricalcium phosphate embedded within a collagen matrix
2D: Two-dimensional
3D: Three-dimensional
