# Supplementary material for: Human platelet-derived extracellular vesicle fractions modulate bone cell metabolism and biologize volume-stable β-TCP matrix in vitro
Source: BMC Med. 2025 Oct 21;23:569. doi: 10.1186/s12916-025-04371-w (PMC12538839; doi:10.1186/s12916-025-04371-w)
Supplement: Supplementary file 1 — Additional file 1: Table 1–Table 1. Murine qRT-PCR-Primer. [file 12916_2025_4371_MOESM1_ESM.docx]

**Table 1. Murine qRT-PCR-Primer**

| **Gene** | **Gene Symbol** | **NCBI Gene ID** | **Primer Sequence (5’-3’ direction)** |
| --- | --- | --- | --- |
| Osteocalcin/bone gamma-carboxyglutamate protein 2 (Bglap2) | *Ocn* | 12097 | Fw CAGACAAGTCCCACACAGCA  Rev CTTGGCATCTGTGAGGTCAG |
| Osteoprotegerin/ Tnfrsf11b tumor necrosis factor receptor superfamily, member 11b | *Opg* | 18383 | Fw CCGAGTGTGTGAGTGTGAGG Rev TGTGTTTCGCTCTGGGGTTC |
| Tumor necrosis factor receptor superfamily, member 11a (Activator of NF-kB) | *Tnfrsf11a/ Rank* | 21934 | Fw TGCAGCTCAACAAGGATACG Rev GTGCAGTTGGTCCAAGGTTT |
| Tumor necrosis factor (ligand) superfamily, member 11 (Tnfsf11)/ Receptor Activator of NF-kB-Ligand | *Rankl* | 21943 | Fw GCAGAAGGAACTGCAACACA Rev GATGGTGAGGTGTGCAAATG |
| Runt-related transcription factor 2 | *Runx2* | 12393 | Fw GCCGGGAATGATGAGAAC Rev GGACCGTCCACTGTCACTT |
| Tartrate-resistant-acid-phosphatase 5 | *Trap* | 11433 | Fw CCAATGCCAAAGAGATCGCC Rev TCTGTGCAGAGACGTTGCCAAG |
| Collagen type I alpha 1 chain | *Col1A1* | 12842 | Fw GAGAGCATGACCGATGGATT Rev TGAGCTCGATCTCGTTGGAT |
| Tumor necrosis factor alpha | *Tnf* | 51926 | Fw ACGGCATGGATCTCAAAGAC Rev GTGGGTGAGGAGCACGTAGT |
| Glyceraldehyde-3-phospate dehydrogenase | *Gapdh* | 14433 | Fw TGTGAACGGATTTGGCCGTA Rev ACTGTGCCGTTGAATTTGCC |
| 40S ribosomal protein S29 | *Rps29* | 6235 | Fw GAAGTTCGGCCAGGGTTCC Rev GAAGCCTATGTCCTTCGCGT |

Primers were used in analysis of osteoblasts and osteoclasts (Fw – forward primer, Rev – reverse primer)
