# Supplementary material for: Human platelet-derived extracellular vesicle fractions modulate bone cell metabolism and biologize volume-stable β-TCP matrix in vitro
Source: BMC Med. 2025 Oct 21;23:569. doi: 10.1186/s12916-025-04371-w (PMC12538839; doi:10.1186/s12916-025-04371-w)
Supplement: Supplementary file 2 — Additional file 2: Figures S1, S2, S3–Fig. S1- Lysates of human platelet lysate derived extracellular vesicle fractionsdo not contain enhanced levels of Leptin and BMP2. Fig. S2–Different hPLEV-F-preparations exert comparable effects on osteoblast mineralization activity. Fig. S3–Under inflammatory conditions, hPLEV-F have no impact on expression levels of bone regulatory proteins in osteoblast. [file 12916_2025_4371_MOESM2_ESM.docx]

Supplements


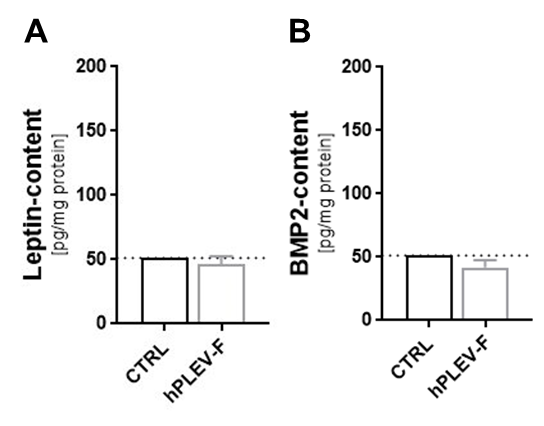


**Figure S1 – Lysates of human platelet lysate derived extracellular vesicle fractions (hPLEV-F) do not contain enhanced levels of Leptin and BMP2.**

hPLEV-F were analysed for protein contents in a multiplex immunoassay. Leptin **(A)** and BMP-2 (Bone morphogenetic protein 2) **(B)** protein levels are shown in pg/mg protein and are compared to an EV-Fraction of pooled human plasma (CTRL); no statistical analyses were performed. Experiments were performed with n=6 for the hPLEV-F analysis and compared to one control value.


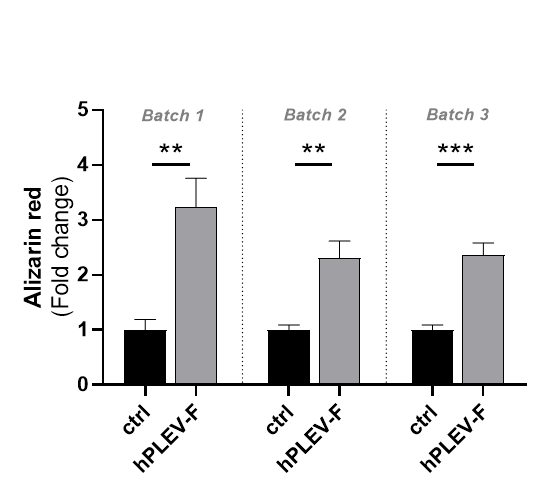


**Figure S2 – Various hPLEV-F-preparations exert comparable effects on osteoblast mineralization activity.**

Bar chart showing mineralisation activity of differentiated murine osteoblasts after 14 days of differentiation without (black) and with hPLEV-F-treatment (grey) by photometric quantification of alizarin red. The experiment was performed with three different hPLEV-F batches. Results are shown as fold change to their respective control in Mean ± S.E.M. Experiments were performed in at least three independent biological replicates. hPLEV-F - human platelet lysate-derived extracellular vesicle fraction; Statistical analyses: Student’s ttest, **p ≤ 0.01

**Figure S3 – Under inflammatory conditions, hPLEV-F have no impact on expression levels of bone-regulatory proteins in osteoblasts.**

Gene expression was analysed in murine osteoblasts differentiated for 5 days that were treated with hPLEV-F for the duration of differentiation and TNFα for the final 24h as an inflammatory trigger. Expression levels of Col1A1 **(A)**, Runx2 **(B)**, Ocn **(C)**, Opg **(D)**, Rankl **(E)** and Tnfα **(F)** are depicted as fold change of ctrl cells without inflammatory trigger (dotted line: ctrl/ctrl) (Shown in Fig. 3A-F). Statistical analyses: Ordinary one-way ANOVA (multiple comparison between ctrl/ctrl, ctrl/hPLEV-F, TNFα/ctrl, TNFα/hPLEV-F) ctrl/ctrl and ctrl/hPLEV-F are depicted in figure 3A-F;
^+^ - compared to ctrl/ctrl; ^#^ - compared to ctrl/ hPLEV-F; ^+^p ≤ 0.05; ^++^p < 0.01; ^+++^p < 0.001; ^++++^p<0.0001; ^#^p ≤ 0.05; ^##^p < 0.01; ^###^p < 0.001; Experiments were performed in three independent biological replicates.
